# Conditional probability and ratio‐based approaches for mapping the coverage of multi‐dose vaccines

**DOI:** 10.1002/sim.9586

**Published:** 2022-09-21

**Authors:** Chigozie Edson Utazi, Justice Moses K. Aheto, Ho Man Theophilus Chan, Andrew J. Tatem, Sujit K. Sahu

**Affiliations:** ^1^ WorldPop, School of Geography and Environmental Science University of Southampton Southampton UK; ^2^ School of Mathematical Sciences University of Southampton Southampton UK

**Keywords:** Bayesian inference, binomial geostatistical model, Demographic and Health Surveys, diphtheria‐tetanus‐pertussis vaccine, vaccination coverage

## Abstract

Many vaccines are often administered in multiple doses to boost their effectiveness. In the case of childhood vaccines, the coverage maps of the doses and the differences between these often constitute an evidence base to guide investments in improving access to vaccination services and health system performance in low and middle‐income countries. A major problem often encountered when mapping the coverage of multi‐dose vaccines is the need to ensure that the coverage maps decrease monotonically with successive doses. That is, for doses i and j, $i<j\Rightarrow p_{i}(s)\geq p_{j}(s)$, where $p_{i}(s)$ is the coverage of dose i at spatial location s. Here, we explore conditional probability (CP) and ratio‐based (RB) approaches for mapping $p_{i}(s)$, embedded within a binomial geostatistical modeling framework, to address this problem. The fully Bayesian model is implemented using the INLA and SPDE approaches. Using a simulation study, we find that both approaches perform comparably for out‐of‐sample estimation under varying point‐level sample size distributions. We apply the methodology to map the coverage of the three doses of diphtheria‐tetanus‐pertussis vaccine using data from the 2018 Nigeria Demographic and Health Survey. The coverage maps produced using both approaches are almost indistinguishable, although the CP approach yielded more precise estimates on average in this application. We also provide estimates of zero‐dose children and the dropout rates between the doses. The methodology is straightforward to implement and can be applied to other vaccines and geographical contexts.

## 1 INTRODUCTION

Many international development goals such as the Sustainable Development Goals (SDGs)^1^ and the Immunization Agenda 2030^2^ recognize the importance of fine‐scale (eg, district level) estimates of health and development indicators (HDIs) for program design, monitoring and evaluation in low‐ and middle‐income countries (LMICs). These estimates help reveal programmatically and epidemiologically important geographic inequities in HDIs, which can often be masked by aggregate national or provincial estimates traditionally produced by most surveys. Thus, the development of model‐based approaches for mapping HDIs has been an active area of research over the last two decades. Maps of HDIs are now routinely produced by the Institute for Health Metrics and Evaluation (IHME),^3^, ^4^, ^5^ the Demographic and Health Surveys (DHS) Program,^6^, ^7^, ^8^ WorldPop^9^, ^10^, ^11^, ^12^, ^13^ and other research groups.

Typically, the data used for map production come from geolocated household surveys, such as the DHS surveys. Bayesian geostatistical modeling techniques^14^, ^15^, ^16^ which leverage geospatial covariate information, usually obtained from a variety of sources, and the spatial dependence between survey clusters are often employed to predict HDIs at unsampled locations, typically over a 1 × 1 km or 5 × 5 km grid covering the area of interest. These high‐resolution maps are also a means to produce estimates of HDIs at more operationally relevant spatial scales, for example, districts, at which estimates can be less uncertain and more interpretable than at the grid square scale.

Geospatial analysis of indicators of childhood vaccination coverage has gained traction in the past few years,^5^, ^11^, ^12^, ^17^, ^18^, ^19^, ^20^, ^21^, ^22^ giving rise to the need for alternative approaches for producing maps of multi‐dose vaccines as often, the maps produced are for single vaccine doses (eg, the first dose of measles‐containing vaccine, MCV1) or the methodology employed when mapping the coverage of multi‐dose vaccines simultaneously (eg, the first and third doses of diphtheria‐tetanus‐pertussis, DTP1 and DTP3) does not consider the relationships between the doses and any challenges these may present.^7^, ^23^ Unlike single vaccination coverage indicators, any modeling framework employed in mapping the coverage of multi‐dose vaccines should guarantee that the coverage maps decrease monotonically with subsequent doses; that is, for vaccine doses i and j, $i<j\Rightarrow p_{i}(s)\geq p_{j}(s)$, where $p_{i}(s)$ is the coverage of dose i at spatial location s. This constraint arises from the fact that it is impossible for the coverage of a subsequent dose of a vaccine to be greater than that of a previous dose. Utazi et al^11^ used a multivariate modeling framework to map the coverage of three doses of DTP vaccine in five study countries. This framework provides a mechanism for leveraging the interdependence between the vaccine doses, but it does not necessarily guarantee that the modeled estimates satisfy the monotonicity constraint. Mosser et al^19^ employed a continuation ratio ordinal regression approach^24^ to enforce this constraint and then applied the approach to model the coverage of DTP1‐3 across Africa. Their approach involved modeling DTP3 coverage (as a reference indicator), defined as probability of receipt of at least three doses (p(d≥3), where d is the number of doses), and two conditional coverage indicators: probability of receipt of two doses given receipt of at most two doses (p(d=2|d≤2)) and probability of receipt of one dose given receipt of at most one dose (p(d=1|d≤1)). These modeled quantities were then used to estimate some intermediate indicators (the probabilities of vaccination with 0,1,2 or ≥3 doses), from which estimates of DTP1 and DTP3 coverage and other quantities of interest were obtained. Mosser et al^19^ noted that the continuation ratio ordinal regression approach was chosen for their work as it allowed the direct modeling of DTP3 which was considered a key indicator in their analysis. However, this approach seems restrictive as it does not enable direct modeling of DTP1 (even in the reverse case of the continuation ratios^25^) which could be a more suitable reference indicator in some contexts. Another limitation of the approach is that fewer data, both in terms of overall sample size and number of sampled locations (compared to one of the approaches explored here), are available to model the conditional coverage quantities owing to their definitions.

Here, we explore a more flexible alternative methodology for mapping multi‐dose vaccines featuring two approaches termed the conditional probability (CP) approach and the ratio‐based (RB) approach. While the RB approach utilizes more robust point‐level data for all modeled indicators unlike the CP approach, both approaches are flexible in terms of choosing either the first or the last dose in the vaccination series as the reference indicator, which is modeled independently. The methodology is embedded within a Bayesian binomial geostatistical modeling framework implemented using the INLA and SPDE approaches. We investigate the effect of varying distributions of point‐level sample sizes on the predictive performance of both approaches using a simulation study. We apply the methodology to mapping the coverage of DTP1‐3 in Nigeria using data from the 2018 Nigeria Demographic and Health Survey.^26^

## 2 METHODOLOGY

### 2.1 The 2018 Nigeria Demographic and Health Survey (NDHS) vaccination coverage data

Georeferenced cluster‐level data on the coverage of each of the three doses of diphtheria‐tetanus‐pertussis vaccine (DTP1‐3) were obtained from the 2018 NDHS.^26^ The survey was designed to be representative at the national and state levels, and for urban and rural areas. A stratified, two‐stage cluster sampling technique was used which involved the selection of clusters (usually enumeration areas) from a national sampling frame in the first stage and households from within the selected clusters in the second stage. Stratification was achieved by separating the administrative level one areas (ie, the 36 states and the Federal Capital Territory) in the country into urban and rural strata, and samples were drawn independently within each stratum.

**FIGURE 1:**
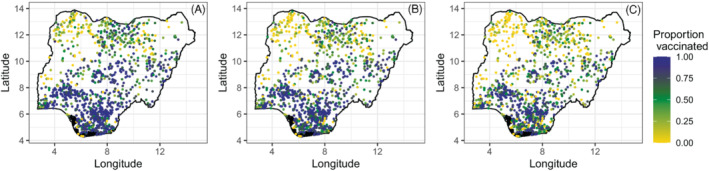
Proportions of children aged 12‐23 months who received (A) DTP1, (B) DTP2, and (C) DTP3 vaccinations at the cluster level

For each cluster location, the information extracted were the number of sampled children aged 12‐23 months, the numbers who had received each of the vaccine doses as evidenced by their vaccination cards or through caregiver recall, and the displaced geographical (ie, longitude and latitude) coordinates of the cluster. Child records with missing vaccination status (ie, the “don't know cases”) were classified as unvaccinated in line with DHS guidelines.^27^ In all, the processed data included 6065 children sampled from 1332 clusters, with 3966 (65.4%), 3513 (57.9%), and 3026 (49.9%) reported to have received DTP1‐3 vaccinations, respectively. Furthermore, the median number of children sampled per cluster was 4 (IQR: 3‐6), and the median numbers of those vaccinated were 3 (IQR: 1‐4), 2(IQR: 1‐4), and 2 (IQR: 1‐3) for each of the respective doses. The empirical cluster‐level coverage is displayed in Figure 1, which shows lower coverage levels in the north compared to the southern areas of the country. We also observe that the spatial distribution of the clusters generally aligns with the population distribution in Nigeria, but there are also areas that were under‐sampled due to insecurity, for example, the northeastern state of Borno.^26^ As in previous work,^12^ our analysis did not include clusters where only one child was sampled. We note that cluster‐level coverage maps of other modeled indicators discussed in the modeling section are provided in supplementary materials.

### 2.2 Geospatial and NDHS‐derived covariate data, processing and covariate selection

As in previous work,^11^, ^12^, ^18^ we assembled some geospatial covariate data known to be either directly linked to coverage or serve as proxies for other unmeasured factors for this study. These include travel time to urban areas, travel time to the nearest health facility (potentially providing routine immunization services), nightlight intensity, and distance to conflict locations as reported in supplementary Table 1. To boost the predictive ability of our models, we obtained additional covariate information from the 2018 NDHS. The geospatial covariates were obtained from various sources and were originally available at different spatial and temporal resolutions. We assembled the most recent data available at the time of analysis, and where applicable, the data were aggregated across multiple years to capture long‐term patterns. All geospatial covariate data were processed using ESRI ArcGIS v10.6 to create standardized 1 × 1 km gridded covariate layers for our study. Further processing using the geospatial covariates was carried out to extract the corresponding data for each cluster location. Following approaches recommended by Perez‐Haydrich et al,^28^ we accounted for the displacement of the cluster locations during covariate data extraction by creating 5 km and 2 km buffers around clusters located in rural and urban areas, respectively. We then extracted the mean values of the continuous covariates using all the grid cells falling within the buffer.

The NDHS‐derived covariates were first calculated at the cluster level using the definitions provided in supplementary Table 2. We then created 1 × 1 km interpolated surfaces of the covariates using kriging interpolation, except the urban‐rural covariate. This was implemented using the “krig” function in the *fields* package^29^ in R, with the optimal range parameter for each covariate determined using a hold‐out cross‐validation exercise. Possible range parameters considered were the quartiles of the distances between the clusters in each state. The selected range parameters were mostly the first or the third quartile of the distances. We elected to use kriging interpolation to create the surfaces of these covariates to avoid introducing the problem of circularity (ie, using the same covariates twice) in the analysis. The gridded surface for the urban‐rural covariate was created using an approach described in Dong and Wakefield,^20^ which utilized gridded population data from WorldPop^30^ and urban population proportion with each administrative level one area within each state obtained from the 2018 NDHS report.^26^

**FIGURE 2:**
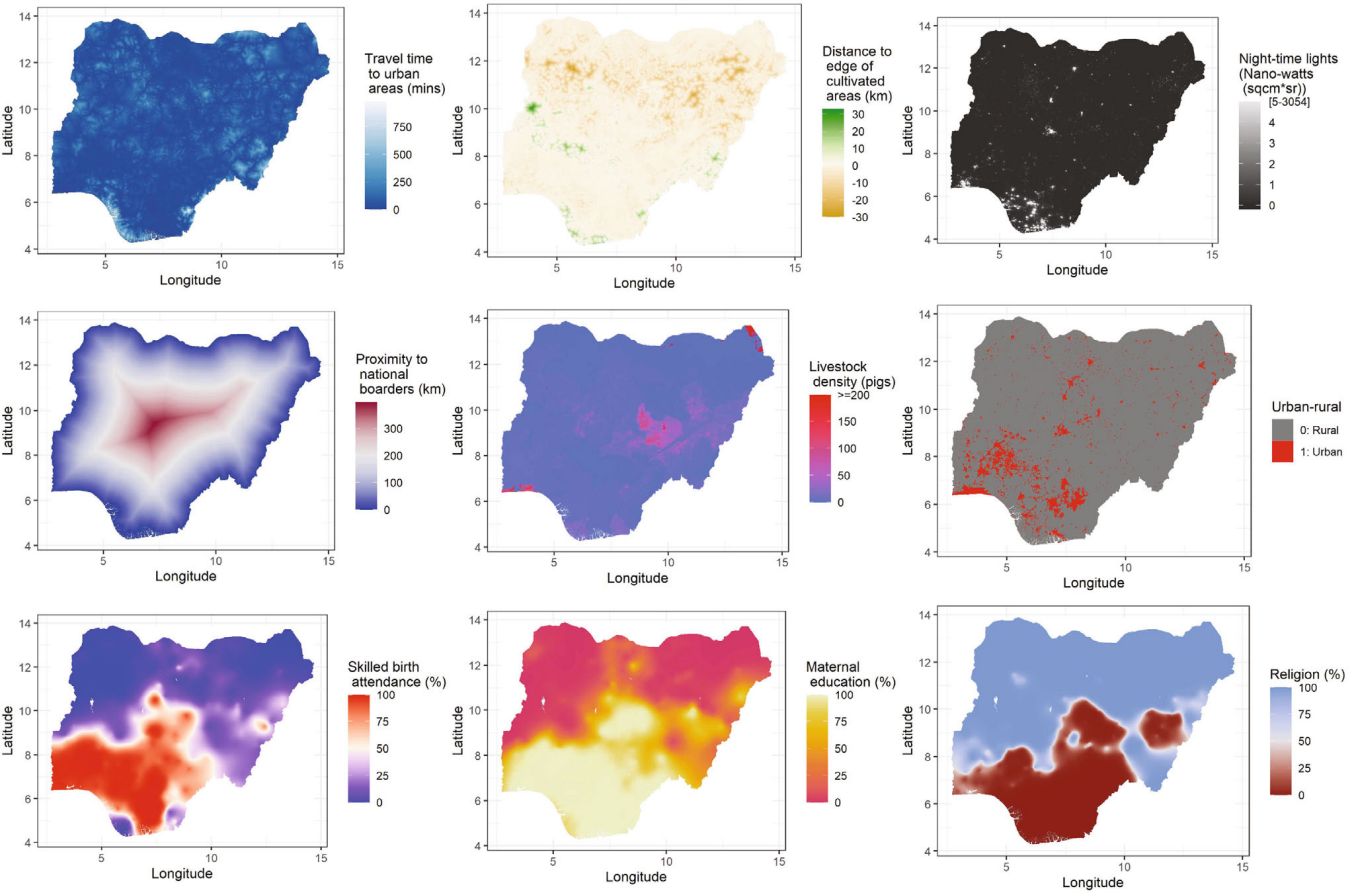
Maps of some geospatial covariates selected for the study

In all, we assembled a total of 23 covariates for the analysis. We selected the best set of covariates for each modeled coverage indicator (see modeling section) in a non‐spatial framework, as is standard practice, using the procedure described in Utazi et al.^18^ We then created a uniform set of covariates for all modeled indicators for each method investigated here. This resulted in a total of 11 covariates included in the analysis as displayed in Figure 2 and supplementary Figure 3 (see supplementary Tables 1 and 2 for details).

### 2.3 Population data

To aggregate the grid‐level predictions of vaccination coverage to different administrative levels (eg, districts, see modeling section) population data were obtained from WorldPop^30^ and processed at 1 × 1 km resolution. These were 2018 estimates of numbers of children aged under 5 years, which we used as a proxy for the 12‐23 month age group. The data were also used to produce the zero dose estimates, that is, estimates of unvaccinated children, through integration with relevant maps of DTP1 coverage.

### 2.4 The proposed method

#### 2.4.1 Bayesian binomial geostatistical model

We begin by specifying a geostatistical model for vaccination coverage. For i=1,…,m, let $y(s_{i})$ denote the number of children vaccinated at cluster location $s_{i}$ out of a total of $n(s_{i})$ children sampled at the location. We assume that $Y(s_{i})|p(s_{i})∼\text{Binomial}(n(s_{i}),p(s_{i}))$, where $p(s_{i})$ is the true vaccination coverage (ie, the proportion of children vaccinated) at location $s_{i}$. Further, $p(s_{i})$ is assumed to follow a logistic regression model given by

(1) $$\text{logit}(p(s_{i}))=x{\left(s_{i}\right)}^{\prime }\beta +\omega (s_{i})+\epsilon (s_{i}),$$

where $x(s_{i})$ is a vector of covariates associated with $s_{i}$, β are the corresponding regression coefficients, $\epsilon (s_{i})$ is an independent and identically distributed (iid) Gaussian random effect with variance, $\sigma_{\epsilon }^{2}$, used to model non‐spatial residual variation, and $\omega (s_{i})$ is a Gaussian spatial random effect used to capture residual spatial correlation in the model. That is, $\omega ={\left(\omega (s_{1}),\ldots ,\omega (s_{n})\right)}^{\prime }∼N(0,\sum_{\omega })$. $\sum_{\omega }$ is assumed to follow the Matérn covariance function^31^ given by

$$\sum_{\omega }(s_{i},s_{j})=\frac{\sigma^{2}}{2^{\nu -1}\Gamma (\nu )}{\left(\kappa ||s_{i}-s_{j}||\right)}^{\nu }K_{\nu }(\kappa ||s_{i}-s_{k}||),$$

where ||·||, denotes the Euclidean distance between cluster locations $s_{i}$ and $s_{j}$, $\sigma^{2}>0$ is the marginal variance of the spatial process, κ is a scaling parameter related to the range $r(r=\frac{\sqrt{8\nu }}{\kappa })$‐the distance at which spatial correlation is close to 0.1, and $K_{\nu }$ is the modified Bessel function of the second kind and order ν>0. Further, for identifiability reasons, we set ν=1, see Lindgren et al.^32^

As noted previously, applying model (1) to map the coverage of multi‐dose vaccines does not guarantee that the monotonic constraint is satisfied for both in‐ and out‐of‐sample predictions. We next explore two alternative approaches to tackle this problem.

#### 2.4.2 The conditional probability (CP) approach

This approach relies on conditional probability rules^16^, ^33^ to express the interdependencies between the coverage indicators and then exploits these to enforce the monotonic constraint. Throughout, we assume a three‐dose vaccination series for simplicity, noting that our approaches can be adapted easily to any number of doses. Let $p_{1}(s)\geq p_{2}(s)\geq p_{3}(s)$ denote the respective coverage of the three‐dose vaccination series at spatial location s. We also refer to these probabilities as the target indicators. All surveyed children at location s can be grouped into four mutually exclusive and completely exhaustive categories, namely zero‐dose children, children who received the first dose but not the second dose, children who received the second dose but not the third dose and children who received all three doses. The corresponding probabilities are denoted using $p_{1^{\prime }}(s),\;p_{1,2^{\prime }}(s),\;p_{2,3^{\prime }}(s)$, and $p_{3}(s)$, respectively. Thus, $p_{1^{\prime }}(s)+p_{1,2^{\prime }}(s)+p_{2,3^{\prime }}(s)+p_{3}(s)=1$. Using conditional probability laws, these probabilities can be further expressed as:

(2) $$\begin{array}{ll} p_{1,2^{\prime }}(s) & =p_{2^{\prime }|1}(s)\times p_{1}(s),\\ p_{2,3^{\prime }}(s) & =p_{3^{\prime }|2}(s)\times p_{2|1}(s)\times p_{1}(s)=p_{3^{\prime }|2}(s)\times p_{2}(s),\\ p_{3}(s) & =p_{3|2}(s)\times p_{2|1}(s)\times p_{1}(s)=p_{3|2}(s)\times p_{2}(s), \end{array}$$

where $p_{2^{\prime }|1}(s)$ is the probability of not receiving the second dose given receipt of the first dose, $p_{2|1}(s)$ is the probability of receiving the second dose given receipt of the first dose, and so on.

Following the progression from $p_{1}(s)$ to $p_{3}(s)$ in Equation (2), it is apparent that the monotonic condition $p_{1}(s)\geq p_{2}(s)\geq p_{3}(s)$ is inherently preserved since $p_{1}(s),\;p_{2|1}(s),\;p_{3|2}(s)\in [0,1]$. Hence, it suffices to model these indicators—$p_{1}(s),\;p_{2|1}(s),\;p_{3|2}(s)$—and then enforce the monotonic constraint through using these modeled indicators to derive the remaining target indicators—$p_{2}(s)$ and $p_{3}(s)$. We note that $p_{3}(s)$ could be used in place of $p_{1}(s)$ as the reference indicator if preferable. Also, the indicators: $\;p_{2|1}(s)$ and $\;p_{3|2}(s)$ are different from the conditional probabilities modeled in the continuation ratio ordinal regression approach,^19^, ^24^ as these do not include cumulative probabilities.

The corresponding point‐level data for the modeled indicators are: $n(s),\;y_{1}(s);n_{1}(s),\;y_{2}(s)$ and $n_{2}(s),\;y_{3}(s)$, for $p_{1}(s),\;p_{2|1}(s)$ and $p_{3|2}(s)$, respectively, where n(s) is the sample size at location s, $y_{1}(s)=n_{1}(s)$ is the number of surveyed children who were reported to have received at least the first dose, $y_{2}(s)=n_{2}(s)$ is the number of children who received at least two doses and $y_{3}(s)$ is the number of children who received the third dose. Observe that $n(s)\geq n_{1}(s)\geq n_{2}(s)$, implying potentially different point‐level sample sizes for these indicators. Given that larger sample sizes tend to reduce prediction error,^12^ smaller values of $n_{1}(s)$ and $n_{2}(s)$ could mean that the conditional probabilities—$p_{2|1}(s)$ and $p_{3|2}(s)$—may not be as well‐estimated as $p_{1}(s)$. This is a potential shortcoming of this approach.

#### 2.4.3 The ratio‐based (RB) approach

The RB approach aims to address the sample size limitation of the CP approach through modeling the ratios of the target indicators as a strategy to enforce the monotonicity constraint. As with the CP approach, there is the flexibility to determine the modeled indicators from either the beginning ($p_{1}(s)$) or the end of the vaccination series ($p_{3}(s)$) and then construct the modeled indicators as ratios of consecutive doses. In our context, the modeled indicators are:

1. The coverage of the first dose $p_{1}(s)$,
2. The ratio of the coverage of the first and second doses $p_{21}(s)=p_{2}(s)\times p_{1}^{-1}(s)$, and
3. The ratio of the coverage of the second and third doses $p_{32}(s)=p_{3}(s)\times p_{2}^{-1}(s)$,

using $p_{1}(s)$ as the reference indicator. From these, $p_{2}(s)$ and $p_{3}(s)$ can be straightforwardly obtained as

(3) $$\begin{array}{ll} p_{2}(s) & =p_{1}(s)\times p_{21}(s)\;\text{and}\\ p_{3}(s) & =p_{2}(s)\times p_{32}(s), \end{array}$$

respectively. Here again, $p_{1}(s),\;p_{21}(s)\in [0,1]$ and $p_{2}(s),p_{32}(s)\in [0,1]$ implies that $p_{1}(s)\geq p_{2}(s)\geq p_{3}(s)$ is satisfied. To obtain the point‐level data for the modeled indicators, let n(s) denote the number of children sampled at location s and $y_{1}(s)$ the corresponding number of children who were reported to have received the first dose. Considering that $p_{21}(s)$ and $p_{32}(s)$ are pseudo indicators, the corresponding pseudo binomial counts can be derived as:

(4) $$\begin{array}{ll} y_{21}(s) & =n(s)\times p_{21}(s)\;\text{and}\\ y_{32}(s) & =n(s)\times p_{32}(s), \end{array}$$

respectively. Observe that the point‐level sample size n(s) is the same for all the modeled indicators. The RB approach is thus unaffected by the potential sample size problem associated with CP approach.

#### 2.4.4 Relationship between the CP and RB approaches

We note that although the CP and RB approaches are different in construction, in our context, the modeled probabilities/indicators are the same under both approaches. Assuming $p_{1}(s)$ to be the reference indicator, it is easy to show that $p_{2|1}(s)=p_{21}(s)$ and $p_{3|2}(s)=p_{32}(s)$, since the probability of receipt of the first and second doses is the same as the probability of receipt of the second dose, and so on. Thus, as highlighted previously, the differences between both approaches lie in the cluster‐level sample sizes associated with the intermediate modeled indicators. Whilst under the RB approach, the cluster‐level sample size is n(s) for both $p_{21}(s)$ and $p_{32}(s)$, the sample sizes for $p_{2|1}(s)$ and $p_{3|2}(s)$ are $n_{1}(s)=y_{1}(s)$ and $n_{2}(s)=y_{2}(s)$, respectively, under the CP approach. In the simulation study in Section 3, we investigate the effect of sample sizes on the predictive performance of both approaches.

### 2.5 Bayesian inference using INLA and SPDE approaches

A fully Bayesian approach was adopted for fitting model (1) for each modeled indicator. Let $\theta =(\beta ,\sigma^{2},r,\sigma_{\epsilon }^{2})$ denote the parameters of the model and z all observe data. The joint posterior distribution of the model can be written as:

(5) $$\begin{array}{ll} \pi (\theta |z) & \propto \overset{m}{\underset{i=1}{\prod }}\{\text{Binomial}(y(s_{i});n(s_{i}),p(s_{i}),\theta ,z)\}\times N(\omega ;0,\sum_{\omega })\times N(\epsilon ;0,\sigma_{\epsilon }^{2}I)\times \pi (\theta ),\\ & \propto \overset{m}{\underset{i=1}{\prod }}\{p{\left(s_{i}\right)}^{y(s_{i})}{\left(1-p(s_{i})\right)}^{n(s_{i})-y(s_{i})}\}\times |\sum_{\omega }{|}^{-\frac{1}{2}}|\;\exp\left(-\frac{1}{2}\omega^{\prime }\sum_{\omega }^{-1}\omega \right)\times \sigma_{\epsilon }^{-m}\;\exp\left(-\frac{\sigma_{\epsilon }^{-2}}{2}\epsilon^{\prime }\epsilon \right)\times \pi (\theta ), \end{array}$$

where π(θ) is the joint prior distribution on θ. We assigned a $N(0,10^{3}I)$ prior to the regression parameter, β. We placed a penalized complexity (PC) prior^34^ on $\sigma_{\epsilon }$ such that $p(\sigma_{\epsilon }>3)=0.01$. Similarly, following Fuglstad et al,^35^ a joint PC prior was placed on the covariance parameters of the spatial random effect, ω. These were: $p(r<r_{0})=0.01$ and p(σ>3)=0.01, with $r_{0}$ chosen to be 5% of the extent of Nigeria in the north‐south direction.

The model was implemented using the integrated nested Laplace approximation—stochastic partial differential equation (INLA‐SPDE) approach.^32^, ^36^ The INLA approach is a faster alternative to the traditional MCMC technique for performing approximate Bayesian inference. The INLA approach produces a numerical approximation of the marginal posterior distributions of each of the unknown quantities in the model. The SPDE approach is particularly required for the estimation of the Gaussian spatial random effect, ω. The approach reduces the computational burden inherent in the estimation of $\sum_{\omega }$ by representing ω as a Gaussian Markov random field (GMRF)—see Lindgren et al.^32^ Further details of the implementation of the INLA‐SPDE approach in our work are provided in supplementary materials.

Given the Bayesian context adopted here, the calculation of the modeled estimates of the remaining target indicators from the modeled indicators was implemented using the posterior samples of the modeled indicators and the formulae provided previously.

All analyses were carried out using R^37^ and R‐INLA package.^38^, ^39^, ^40^

### 2.6 Model validation

For each approach, the performance of the fitted models for out‐of‐sample prediction using the modeled indicators was assessed at the cluster level using a k‐fold cross‐validation scheme, with the folds created as random splits of the n cluster locations. We set k=10 and using the observed (p(s)) and predicted ($\hat{p}(s)$) coverage levels for $m_{c}$ validation locations, we computed the following model evaluation metrics:

$$\begin{array}{llll} \text{Average bias, AvBias} & =\frac{1}{m_{c}}\overset{m_{c}}{\underset{i=1}{\sum }}(\hat{p}(s_{i})-p(s_{i})),\; & & \\ \text{Root mean square error, RMSE} & =\sqrt{\overset{m_{c}}{\underset{i}{\sum }}{\left(\hat{p}(s_{i})-p(s_{i})\right)}^{2}/m_{c}},\; & & \end{array}$$

and the correlation between observed and predicted values were used to evaluate predictive performance. All three metrics assess the accuracy of the point predictions. The smaller the AvBias (in absolute value) and RMSE, the better the predictions. Conversely, the higher the correlation, the better the predictions. These metrics were calculated and averaged over the cross‐validation folds.

Additionally, for the target indicators, the modeled estimates were compared with the direct survey estimates (often considered to be the gold standard^41^) at the state level as in previous work.^12^

### 2.7 Prediction

For both the CP and RB approaches, predictions using model (1) were first produced at 1 × 1 km resolution for the target indicators (ie, $p_{1}(s),p_{2}(s)$, and $p_{3}(s)$). Administrative‐level predictions using the model were obtained as population‐weighted averages taken over all the grid cells falling within each administrative unit. That is, for area $A_{i}(i=1,\ldots ,m_{A}$ areas, eg, districts), vaccine dose k, and posterior sample r,

$$p_{k}^{r}(A_{i})=\int_{A_{i}}p_{k}^{r}(s)\times q(s)ds\approx \sum_{j=1}^{m_{i}}p_{k}^{r}(s_{j})\times q(s_{j}),$$

where $m_{i}$ is the number of grid locations with centroids in are $A_{i}$ and q(s) is the proportion of the population of the area at grid location s.

To compare the prediction uncertainties associated with the approaches being investigated, we computed the average prediction variance (APV) which is given by

$$\text{APV}=\int_{A}\mathrm{Var}\{p_{k}(s)|z\}ds\approx \frac{1}{m_{p}}\overset{m_{p}}{\underset{i=1}{\sum }}\mathrm{Var}\{{\hat{p}}_{k}^{i}(s)|z\},$$

where $m_{p}$ is the number of prediction grid cells. Predicted maps with lower APVs are often desirable.

## 3 SIMULATION STUDY

Here, we describe a simulation study undertaken to investigate the effect of varying point‐level sample sizes on the predictive performance of the CP and RB approaches. Using Nigeria as an example geography and the survey clusters from the processed data described in Section 2.1 as the observation locations, data were simulated from model (1) using the following true parameter values: $\sigma^{2}=1,\;r=2.62,\;\sigma_{\epsilon }^{2}=1$ and $\beta ={\left(0.5,0.8,0.8,0.2\right)}^{\prime }$ corresponding to a covariate vector with an intercept term and three variables simulated from N(0,1),Gamma(1,1) and t(2).

We note that the value of r corresponds to the first quartile of the distances between the observation locations. Also, we take the 5 × 5 km grid covering the entire country to be the prediction locations for faster computation. Once we have simulated the values of $p_{1}(s)$ for both the observation and prediction locations, we proceeded to simulate those of $p_{2}(s)$ and $p_{3}(s)$ by adding an incremental parameter to the right‐hand side of Equation (1) each time to reflect changes in either the regression part of the model or the residual terms. This also ensures that the simulated values of all three indicators satisfy the monotonicity constraint $p_{1}(s)\geq p_{2}(s)\geq p_{3}(s)$. Mimicking the patterns in the vaccination coverage data described in Section 2, we set the incremental parameter equal to −1.3 for $p_{2}(s)$ and −2.5 for $p_{3}(s)$. Next, we assumed the following discrete uniform distributions for the sample sizes at the observation locations: U{2,10},U{2,20},U{2,30},…,U{2,80} to reflect varying ranges of sample sizes, although we note that in most DHS surveys, cluster level sample sizes >30 are uncommon. These larger sample sizes are therefore included in the study mainly for illustrative purposes. After obtaining the corresponding counts of successes $y_{1}(s),\;y_{2}(s)$, and $y_{3}(s)$ through multiplying the sample sizes by the simulated probabilities to preserve the monotonicity constraint, we then proceeded to calculate the additional indicators required for both the CP and RB approaches. This simulation set up resulted in three true 5 × 5 km coverage maps for the target indicators: $p_{1}(s),\;p_{2}(s)$, and $p_{3}(s)$, and a total of 24 data sets, each comprising the same true coverage levels at the observation locations for the target indicators but different sample size distributions. The simulated point and grid level data for the target indicators are displayed in supplementary Figures 4 and 5.

Additionally, we considered a second scenario in which we assumed that the data were not spatially correlated. The data used to investigate the predictive performance of the CP and RB approaches in this case were simulated using the same study design as before, but excluding the spatial random effect, ω, from the model.

For each simulation scenario and modeling approach, we analyzed the simulated data using the Bayesian approaches described in Section 2.5, placing similar prior distributions on all the parameters of the model. We evaluated the predictive performance of both approaches using the metrics described in Section 2.6, all of which were calculated using the true and predicted 5 × 5 km maps of $p_{1}(s),\;p_{2}(s)$, and $p_{3}(s)$ (out‐of‐sample validation) and the corresponding point‐level data for both the modeled indicators and target indicators (in‐sample validation) in each case. Additionally, to gain more insights into the predictive performance of the approaches, we calculated the mean absolute error (MAE $=\sum_{i=1}^{m_{p}}|\hat{p}(s)-p(s_{i})|/m_{p}$) and the actual coverage of the 95% prediction intervals (95% coverage $=100\times \sum_{i=1}^{m_{p}}I({\hat{p}}_{l}(s_{i})\leq p(s_{i})\leq {\hat{p}}_{u}(s_{i}))/m_{p}$), where $m_{p}$ is the number of prediction locations, $p(s_{i})$ is the true/simulated coverage at location $s_{i}$ and $\hat{p}(s_{i})$ is the corresponding predicted coverage, ${\hat{p}}_{l}(s_{i})$ and ${\hat{p}}_{u}(s_{i})$ are the lower and upper limits of the prediction intervals respectively, and I(·) is an indicator function. The MAE is also used to evaluate the accuracy of the point predictions while the achieved 95% coverage evaluates the accuracy of the uncertainties associated with the predictions. The lower the MAE, the better the prediction. Also, the closer the achieved coverage is to the true value of 95%, the better the predictions.

**FIGURE 3:**
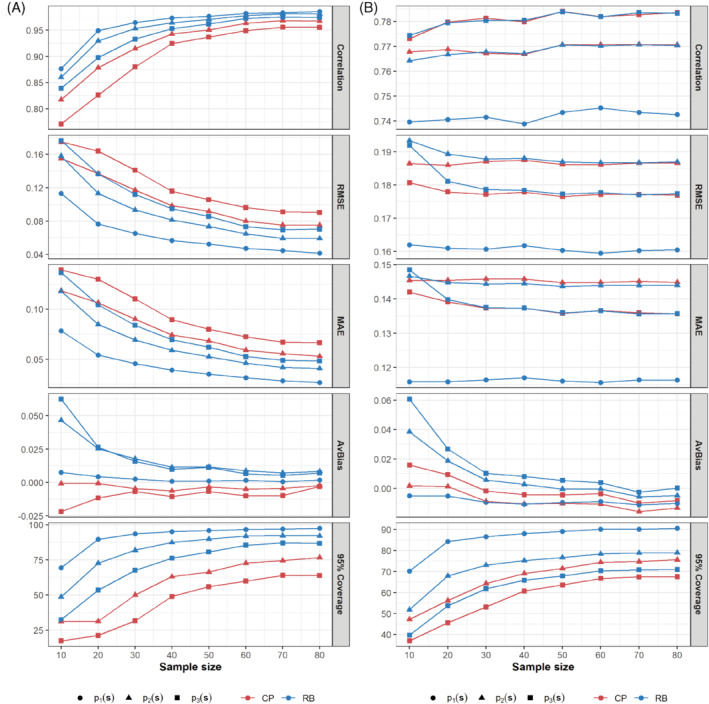
Predictive performance of the conditional probability (CP) and ratio‐based (RB) approaches based on different sample size distributions for spatially‐correlated point‐level data: (A) In‐sample prediction of the target indicators; (B) out‐of‐sample prediction of the target indicators over a 5 × 5 km grid

The results we obtained are displayed in Figure 3 and supplementary Figures 6‐9. Figure 3A clearly shows that for in‐sample prediction, predictive performance improved as sample sizes increased, and based on nearly all the metrics (except AvBias), the RB approach clearly performed better than the CP approach. This is an indication that the modeled indicators were generally better estimated under the RB approach (see supplementary Figure 6), likely due to the availability of larger sample sizes at the point level for these indicators when using this approach. Also, we observe that for both approaches, the point estimation of $p_{1}(s)$ (this indicator is the same for both approaches in the simulation design, hence the overlaps in the figures) appears to be consistently better than those of other target indicators. This validates our earlier speculation that the reference indicator—$p_{1}(s)$, which is modeled directly and independently, is likely to be more robustly estimated than other target indicators. Similar patterns were also observed in the in‐sample prediction of the modeled indicators under each approach as shown in supplementary Figure 6.

However, for out‐of‐sample prediction based on the 5 × 5 km grid points, Figure 3B shows that the effect of sample size is negligible when examining the correlation, RMSE and MAE statistics, with both approaches having very similar performances in these instances. Nevertheless, when examining the AvBias and 95% coverage, both approaches exhibit better predictive performance with increasing sample size, particularly for sample sizes ≤50. Also, in terms of 95% coverage, the RB approach is consistently the better approach for all sample sizes; whereas based on AvBias, the CP approach is better than the RB approach for sample sizes ≤20. The former case is likely an artefact of the larger point level sample sizes for the modeled indicators in the RB approach.

Additionally, owing to the dependence of the sample sizes for $p_{2|1}(s)$ and $p_{3|2}(s)$ on the values of $p_{1}(s)$ in the CP approach, we investigated the predictive performance of both approaches when $p_{1}(s)\leq 0.3$, as this is likely to yield very small sample sizes for both conditional probabilities in the CP approach. Interestingly, the results we obtained (see supplementary Figure 7) are very similar to the results reported in Figure 3 for the full range of values of $p_{1}(s)$, except that the AvBias estimates for both approaches are very close in both in‐sample and out‐of‐sample predictions for all sample sizes. Also, we obtained very similar results to those shown in Figure 3 in an additional sensitivity analysis using smaller sample size distributions, that is, discrete uniform U{2,8},U{2,10},U{2,12},U{2,15},U{2,20},…,U{2,35} (see supplementary Figure 8).

In general, these results reveal that the effect of sample size on both approaches is more pronounced in in‐sample prediction, in which case the RB approach outperformed the CP approach. For out‐of‐sample prediction, no approach is uniformly the better approach, and the effect of sample size appears to matter for bias and uncertainty estimation only.

With spatially uncorrelated data, the patterns in the results (see, eg, supplementary Figure 9) are similar to those shown in Figure 3, hence we do not discuss these further.

## 4 RESULTS OF ANALYSIS OF THE 2018 NIGERIA DEMOGRAPHIC AND HEALTH SURVEY (NDHS) VACCINATION COVERAGE DATA

Here, we present the results of application of the proposed approaches to mapping DTP1‐3 vaccination coverage using the 2018 NDHS data, including the dropout rates between the doses and estimates of zero‐dose children.

With the CP approach, the covariates chosen for model‐fitting and prediction were: maternal education, skilled birth attendance, livestock (pigs) density, proximity to national borders, urbanicity (ie, urban/rural), religion, night‐time lights, and household wealth. For the RB approach, the first five covariates were also selected in addition to travel time to urban areas, distance to the edge of cultivated areas and distance to conflict areas.

For both approaches, estimates of parameters of the fitted models are reported in supplementary Tables 3 and 4 for the modeled indicators. For the CP approach, maternal education, religion, and skilled birth attendance were significant predictors of DTP1 coverage (ie, $p_{1}(s)$). Maternal education was also a significant predictor of $p_{2|1}(s)$, while skilled birth attendance was a significant predictor of $p_{3|2}(s)$. For the RB approach, similar patterns were also observed in the relationships between the modeled indicators and the covariates. Maternal education, and skilled birth attendance were significant predictors of $p_{1}(s)$. Maternal education was also the only significant predictor of $p_{21}(s)$, while skilled birth attendance and livestock density (pigs) were significant predictors of $p_{32}(s)$. Further, education and skilled birth attendance had positive relationships with coverage while religion had a negative relationship with coverage in all cases as expected. Interestingly, livestock (pigs) density also had a positive relationship with coverage. We note that the regression coefficients associated with these significant covariates can be exponentiated to quantify the effect of a unit increase in these covariates on the odds of vaccination. However, this is not of interest here for various reasons including our focus on prediction and potential aggregation bias that could occur with some of the covariates that can be measured at the individual child level. For the CP approach, the estimated spatial ranges were between 115 and 239 km, whereas for the RB approach, these were between 111 and 224 km for the modeled indicators in each case (see supplementary Tables 3 and 4).

Cluster‐level out‐of‐sample model validation results are presented in Table 1. These results show the predictive performance of the fitted models for equivalent modeled indicators under both approaches. Very similar results were obtained for $p_{1}(s)$ with both approaches, even though different sets of covariates were used in the analysis. For the last two indicators, the RB approach had consistently lower AvBias while the CP approach had consistently lower RMSE values. Mixed results were obtained when considering the correlation statistics. Thus, no approach produced consistently better results for these equivalent modeled indicators.

**TABLE 1:** Model validation statistics based on a k‐fold cross‐validation exercise and average prediction variance estimates

Modeled indicators	AvBias	RMSE	Correlation	Target indicators	APV
Conditional probability approach					
$p_{1}(s)$	−0.001	0.220	0.740	$p_{1}(s)$	0.020
$p_{2|1}(s)$	0.005	0.196	0.395	$p_{2}(s)$	0.021
$p_{3|2}(s)$	0.005	0.219	0.322	$p_{3}(s)$	0.023
Ratio‐based approach					
$p_{1}(s)$	−0.001	0.220	0.739	$p_{1}(s)$	0.022
$p_{21}(s)$	0.002	0.218	0.357	$p_{2}(s)$	0.034
$p_{32}(s)$	0.001	0.258	0.356	$p_{3}(s)$	0.051

When considering the uncertainties in the modeled 1 × 1 km estimates of the target indicators, the APV values show that that the CP approach outperformed the RB approach. Also, the directly modeled indicator, $p_{1}(s)$, had the lowest APV of all the three target indicators in each case, which is a further indication that this indicator was more robustly estimated, the evidence of which is stronger under the RB approach. Subsequent results presented in this work are therefore based on the CP approach, with comparisons with the RB approach included where necessary.

Lastly, in supplementary Figure 10, we further validate the estimates of the target indicators using the direct survey estimates at the state level. These plots indicate that there is a strong correspondence (correlation ≥0.94 in each case) between the direct and modeled estimates both when considering the CP and RB approaches. The plots also show that the uncertainties associated with the RB approach were generally wider than those of the CP approach, further corroborating the results presented in Table 1.

### 4.1 DTP1‐3 coverage maps

**FIGURE 4:**
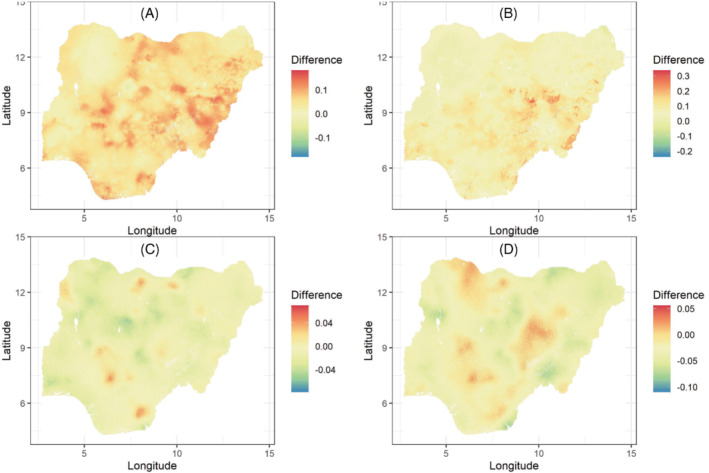
Differences between 1 × 1 km predicted maps of DTP2 (A, C) and DTP3 (B, D) obtained through using the conditional probability and ratio‐based approaches when covariates were included (A, B) and excluded (C, D) from the fitted models

We first compare 1 × 1 km predicted maps of DTP2 and DTP3 coverage produced using the CP and RB approaches in Figure 4. Panels (A) and (B) show slight differences between the predicted maps when covariates were included in the fitted models. These differences became narrower when covariates were excluded from the analysis as shown in panels (C) and (D). Hence, these maps demonstrate that both approaches produced very similar grid level predictions despite being different in construction and implementation.

**FIGURE 5:**
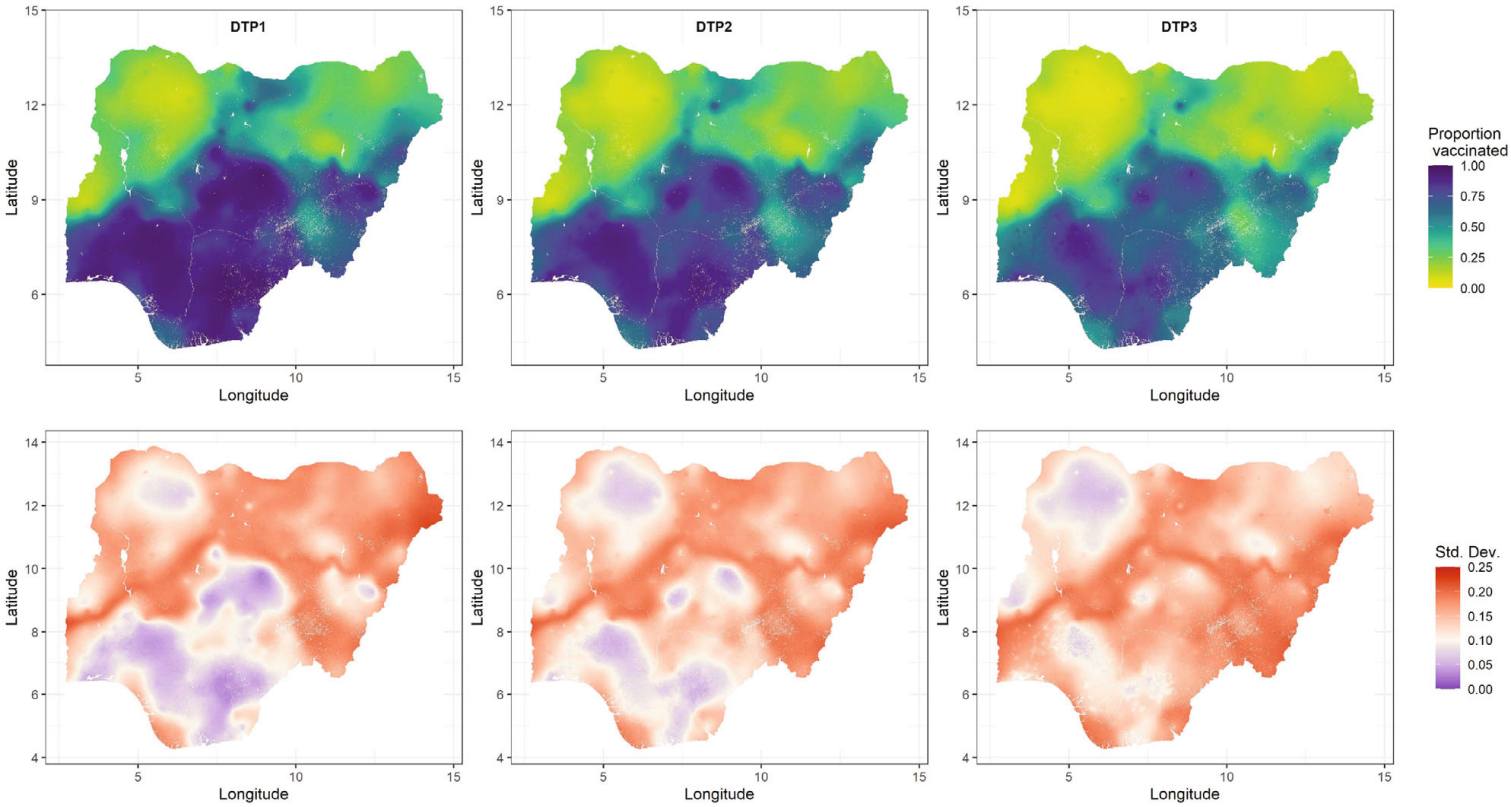
Predicted 1 × 1 km maps of DTP1‐3 coverage and associated uncertainties shown as standard deviations

In Figure 5 we present the coverage maps of all three doses produced using the CP approach. There are substantial heterogeneities in the coverage of each dose, with coverage levels markedly higher in the south compared to the north, particularly the northeastern and northwestern areas. Coverage can also be seen to generally decrease when progressing from DTP1 to DTP3, as expected. The “smooth” predicted maps are most likely an artefact of the kriged DHS covariates (see, eg, Figure 2) which were mostly significant predictors of coverage in the fitted models. The uncertainties associated with these estimates, presented as standard deviations, show that for DTP1, the southern areas where higher coverage levels were estimated had lower uncertainty compared to the north. Some lower coverage areas in the northwest were also predicted with lower uncertainty. Similar patterns are apparent in the coverage maps of DTP2 and DTP3, although there are more areas of higher uncertainty. Generally, areas with lower density of cluster locations (see Figure 1) tend to have higher uncertainty. Also, the patterns in these uncertainty estimates are likely due to the binomial likelihood used in the model—estimates close to the endpoints of the unit interval tend to have higher precision than estimates lying close to the middle of the interval.^18^ At the district or local government area (LGA) level (see supplementary Figure 11), significant inequalities in coverage still exist and patterns in coverage are generally similar to those shown in Figure 5.

### 4.2 Dropout rates and zero‐dose estimates

**FIGURE 6:**
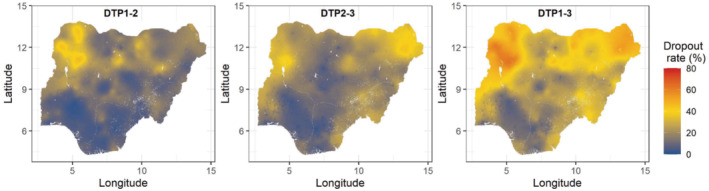
Dropout rates between the doses at 1 × 1 km resolution

Maps of relative dropout rates between the doses (calculated as $100\times ({\hat{p}}_{i}(s)-{\hat{p}}_{j}(s))/{\hat{p}}_{i}(s);i<j$) are shown in Figure 6. Clearly, the dropout rates are generally higher between DTP2 and DTP3 (DTP2‐3) than between DTP1 and 2 (DTP1‐2). Also, lower dropout rates were estimated in areas with higher coverage, while higher dropout rates were estimates in areas with lower coverage. These patterns are more evident when examining the dropout rates between DTP1 and DTP3. This suggests that factors responsible for high dropouts may also influence the likelihood of receipt of DTP1. There are also visible spots of areas of lower dropout rates in urban areas.

**FIGURE 7:**
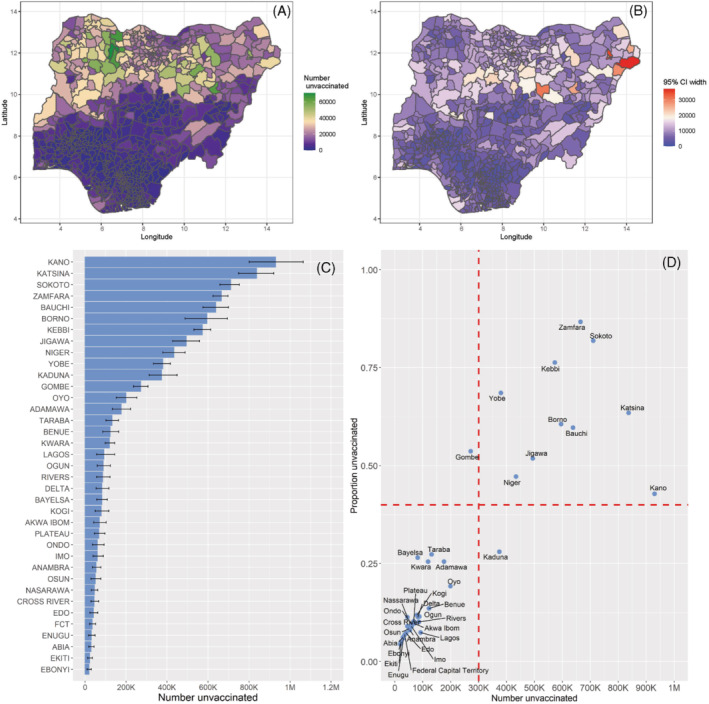
Estimates of numbers of zero‐dose children aged under 5 years and associated uncertainties at the district (A, B) and state levels (C). The relationship between proportions of unvaccinated children and corresponding zero‐dose estimates at the state level is shown in panel (D). The red dotted lines are used to show different prioritization scenarios

Estimates of numbers of children aged under 5 years who had not received any DTP doses, that is, zero‐dose children, are displayed at both the district and state levels in Figure 7 (see supplementary Table 5 for details of the district‐level estimates). These zero‐dose estimates were produced using relevant administrative level coverage estimates and associated uncertainties, and population estimates which were assumed to be fixed. An alternative approach for producing the zero‐dose estimates is to use relevant grid level coverage and population estimates and then aggregate the resulting zero‐dose estimates to administrative levels of interest. While both approaches can produce very similar zero‐dose (point) estimates, the second approach could sometimes yield unreasonably wide uncertainty intervals. Districts with the most unvaccinated children are located in Zamfara (Bungudu, Zurmi, Gusau, Kaura Namoda, Maradun, Maru), Gombe (Yamaltu/Deba), Bauchi (Darazo, Bauchi, Ningi), Kebbi (Wasagu/Danko), Yobe (Fune), Sokoto (Dange‐Shuni), Borno (Jere), and Jigawa (Birnin Kudu) states, all of which had at least 50 000 zero‐dose children. The uncertainties associated with these estimates (Figure 7B) are generally low (95% CI width <20000), apart from some districts in the northeastern and northwestern areas where higher uncertainties were estimated. The patterns in the uncertainty estimates generally reflect the patterns in the uncertainties in the underlying district‐level estimates presented in supplementary Figure 11, as expected.

At the state level, Kano, Katsina, Sokoto, Zamfara, Bauchi, Borno, Kebbi, Jigawa, and Niger states had at least 400 000 zero‐dose children. These are mostly states where districts with higher estimates of numbers of zero‐dose children were located (panel A), and where the poorest coverage levels were estimated. The uncertainties associated with the zero‐dose estimates appear to increase as the estimates increase, and these generally show that the numbers of zero‐dose children were reasonably well estimated. Furthermore, states where there is an intersection of lower to moderate coverage and higher zero‐dose estimate, as shown in panel (D) should be considered as priority areas for improvements in routine immunization coverage.

## 5 DISCUSSION

In this article, we have explored alternative approaches for mapping the coverage of multi‐dose vaccines at fine spatial scales in low‐ and middle‐income settings. Both approaches examined are flexible in terms of using either the first or the last dose in the vaccination series as the reference indicator, which can often be an important consideration. Furthermore, one of the approaches—the RB approach—is not subject to potential sample size restrictions that can be encountered when modeling conditional probabilities which are used to induce the monotonicity constraint in some approaches (eg, the CP approach explored here and the continuation ratio ordinal regression approach used in Mosser et al^19^). We illustrated this using a simulation study in which we found out that the RB approach consistently performed better than the CP approach for in‐sample prediction under varying point‐level sample size distributions. We also noted that increasing point‐level sample sizes had marked positive impact on in‐sample prediction using both approaches. However, for out‐of‐sample prediction, no approach was consistently the better approach. Also, in this case, increases in point‐level sample sizes mainly led to improvements in bias and uncertainty estimation. We, however, note that although increasing the point‐level sample sizes is desirable in a geostatistical context, in practice, this may need to be balanced against design‐based large‐area survey analysis considerations, where larger cluster‐level sample sizes can be statistically inefficient.^12^

We applied the methodology to map the coverage of DTP1‐3 in Nigeria using data from the 2018 NDHS. We modeled DTP1 as the reference indicator due to our interest in producing estimates of zero‐dose children. We demonstrated that both approaches yielded very similar results for this application. Our maps of DTP1 and DTP3 coverage—both of which are often used to evaluate access to routine immunization (RI) services and the general performance of RI programs—produced some interesting patterns.^11^ These maps revealed substantial heterogeneities in coverage as well as a characteristic north‐south divide.^12^, ^20^ The northeast, the northwest and parts of the north central zones of the country are the problematic areas where efforts should be targeted to fill coverage and immunity gaps. In addition, the patterns in the dropout rates suggest that areas with lower coverage were more likely to have higher dropout rates, as also noted in a previous study.^11^ This is an indication that factors responsible for non‐vaccination in these areas are likely responsible for the failure to complete the vaccination series. Aheto et al^42^ found these factors to include non‐ownership of a health card/document, non‐receipt of vitamin A (both are indicators of access to health/vaccination services), poor maternal education, religion and maternal age (being born to a younger mother), some of which were included as covariates in this study. Thus, any strategies geared towards improving RI coverage in the country should aim to address the inequities emanating from these factors. The process of prioritizing subnational areas for RI improvements often involves an assessment of estimates of DTP vaccine coverage and corresponding numbers of DTP zero‐dose children, local measles epidemiology and other concomitant factors such as insecurity and prevalence of other childhood diseases. The coverage maps and zero‐dose estimates presented here can serve as a useful input into this process to guide the allocation of resources at the national and subnational levels, as well as being credible alternatives to administrative coverage estimates whose utility is often limited by numerator and denominator issues.^43^

Our work is subject to some limitations. With both approaches, some of the modeled indicators were not as robustly estimated as the reference indicator in our application. This may have been an effect of the little variation in these indicators (supplementary Figures 1 and 2), which also meant that they were more difficult to predict. The data we analyzed included information on vaccination coverage obtained from vaccination cards and through caregiver recall. Although, this increases the data available for modeling, it has the potential to introduce recall bias in the analysis. Grid‐level and aggregated predictions of vaccination coverage (including comparisons with direct survey estimates) and corresponding zero‐dose estimates can be influenced by the covariates included in the analyses—see, for example, Giorgi et al.^44^ Our analyses included both geospatial and NDHS‐derived covariates, but our results showed that the latter appeared to have suppressed the geospatial covariates. We were unable to investigate the effect of this outcome and the contributions of both sets of covariates (both separately and combined) to the predictions, but we plan to undertake this elsewhere. Furthermore, we were unable to account for the uncertainties associated with the population estimates used in producing the zero‐dose estimates. Accounting for the uncertainties in both the population and coverage estimates simultaneously when producing zero‐dose estimates will be better implemented in a joint modeling framework, which will constitute part of future work. In our application, we chose the CP approach because it yielded smaller APV values for the target indicators. While this choice is plausible in a geospatial analysis context in which estimates with less uncertainties are desirable, we note that the APV metric does not evaluate the accuracies of the uncertainties estimated by both approaches. Also, estimates produced in under‐sampled areas, for example, conflict areas in Borno state, could be biased if the relationships between the covariates and vaccination coverage in those areas were different from those of other areas where data were collected. Lastly, our methodology and application focused on a snapshot in time. Additional insights can be gained from analyzing trends in coverage over time. In future work, we will consider an extension of the methodology to the spatiotemporal setting.

In conclusion, we consider this work a useful addition to the growing body of methodology for producing maps of vaccination coverage. It is straightforward to apply the methodology to map the coverage of other multi‐dose vaccines, for example, the pneumococcal conjugate vaccine and rotavirus vaccine, even as efforts within the global health community are continually targeted towards improving vaccination services, improving access to new vaccines and accelerating progress towards disease elimination.

## FUNDING INFORMATION

This work was supported by funding from the Bill and Melinda Gates Foundation (Investment ID INV‐003287). Chigozie Edson Utazi and Andrew J. Tatem received the Grant. The funder did not play any role in the study design, data collection, analysis and interpretation of data, the report writing, and the decision to submit the manuscript for publication.

## CONFLICT OF INTEREST

The authors declare that they have no known competing financial interests or personal relationships that could have appeared to influence the work reported in this article.

## Supporting information


**Data S1**: Supporting Information

## Data Availability

DHS data supporting this study are publicly available from https://dhsprogram.com/data/available‐datasets.cfm. Other data are publicly available via the sources referenced in the methods section. These can also be obtained from the authors upon request. R scripts used for the analyses are available on GitHub (https://github.com/EdsonUtazi/Code‐for‐multi‐dose‐vax‐paper‐v2)
