# Supplementary material for: Conditional probability and ratio‐based approaches for mapping the coverage of multi‐dose vaccines
Source: Stat Med. 2022 Sep 21;41(29):5662–78. doi: 10.1002/sim.9586 (PMC9826002; doi:10.1002/sim.9586)
Supplement: Supplementary file 1 — Data S1: Supporting Information [file SIM-41-5662-s001.pdf]

Conditional probability and ratio-based approaches for mapping the coverage of multi-dose vaccines

CE Utazi[1,2*], JMK Aheto[1], HMT Chan[1,2], AJ Tatem[1], SK Sahu[2]

[1]WorldPop, School of Geography and Environmental Science, University of Southampton, Southampton, SO17 1BJ, UK.

[2] Mathematical Sciences, University of Southampton, Southampton, SO17 1BJ, UK

*Corresponding author
E-mail: c.e.utazi@soton.ac.uk

Supplementary information

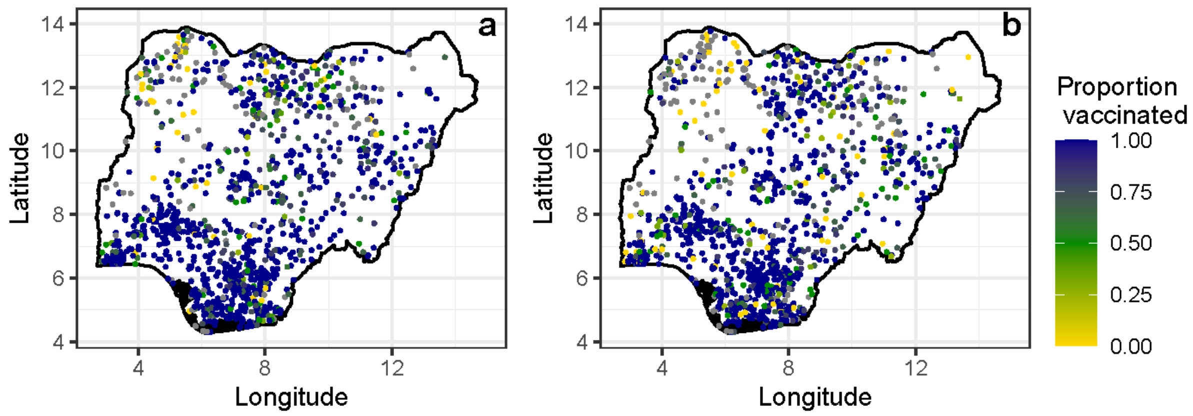

Figure 1: Conditional probability approach: Proportions of children aged 12-23 months (a) who received DTP2 following receipt of DTP1 ($p_{2|1}$) and (b) who received DTP3 following receipt of DTP2 ($p_{3|2}$).

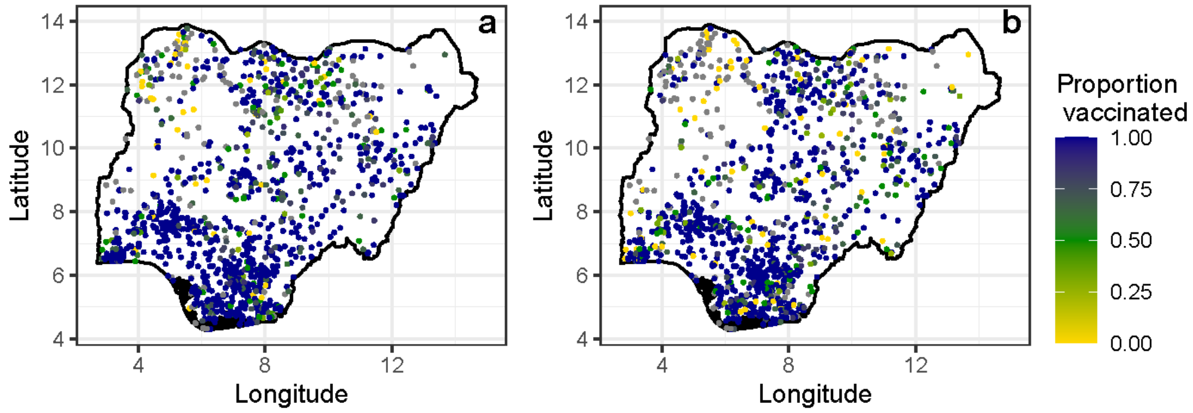

Figure 2: Ratio-based approach: Ratios of proportions of children aged 12-23 months (a) who received DTP1 and DTP2 (p_{21}) and (b) DTP2 and DTP3 (p_{32}).

Table 1: A description of the geospatial covariates considered in the analysis

Covariate	Unit/Description	Type	Source
Livestock density (cattle, chicken, goats, pigs, sheep)	No of cattle, chicken, goats, pigs, sheep per sq. km	Continuous	Gilbert, M. *et al.* (2018) Global Distribution Data for Cattle, Buffaloes, Horses, Sheep, Goats, Pigs, Chickens and Ducks in 2010. Nature Scientific data, 5:180227. doi: 10.1038/sdata.2018.227
Distance to the edge of cultivated areas	Kilometres	Continuous	ESA (European Space Agency) CCI (Climate Change Initiative) Land Cover project 2017. "Land Cover CCI Product - Annual LC maps from 2000 to 2015 (v2.0.7)." http://maps.elie.ucl.ac.be/CCI/viewer
Travel time to urban areas (where population >=50,000)	Minutes	Continuous	Weiss, D.J. *et al*. (2018). A global map of travel time to cities to access inequalities in accessibility in 2015. *Nature*.
VIIRS Nightlight intensity	Nano-watts (sqcm*sr)	Continuous	NOAA – Visible Infrared Imaging Radiometer Suite. https://ngdc.noaa.gov/eog/viirs/index.html (2016)
Distance to conflicts	Kilometres	Continuous	*[Derived from]* Raleigh, Clionadh, Andrew Linke,

			Håvard Hegre and Joakim Karlsen. (2010). "Introducing ACLED-Armed Conflict Location and Event Data." Journal of Peace Research 47(5) 651-660.
Average aridity index (2013-2018)	-	Continuous	CGIAR-CSI Global-Aridity and Global-PET Database. Available at https://cgiarcsi.community/data/global-aridity-and-pet-database/
Average precipitation (2013-2018)	mm/month	Continuous	Harris I, Osborn TJ, Jones P and Lister D (2020) Version 4 of the CRU TS Monthly High-Resolution Gridded Multivariate Climate Dataset. Scientific Data (https://doi.org/10.1038/s41597-020-0453-3).
Average number of wet days (2013-2018)	Number of days	Continuous	Harris I, Osborn TJ, Jones P and Lister D (2020) Version 4 of the CRU TS Monthly High-Resolution Gridded Multivariate Climate Dataset. Scientific Data (https://doi.org/10.1038/s41597-020-0453-3).
Proximity to national borders	Kilometres	Continuous	[derived from] Global Administrative Areas (2021). GADM database of Global Administrative Areas, version 3.6. [online] URL: www.gadm.org.
Average MODIS Enhanced Vegetation Index (EVI) 2013-2018	-	Continuous	Didan, K. (2015). MOD13A3 MODIS/Terra vegetation Indices Monthly L3 Global 1km SIN Grid V006. NASA EOSDIS LP DAAC.
Average MODIS daytime land surface temperature 2013-2018	Degree Celsius	Continuous	Wan, Z. *et al*. MOD11C3 MODIS/Terra Land Surface Temperature/Emissivity Monthly L3 Global 0.05Deg CMG Voo6 [Data set]. NASA EOSDIS Land Processes DAAC.
Slope index	Average SRTM Slope in degrees		Lloyd, Christopher T. (2017) High resolution global gridded data for use in population studies. In *The International Archives of the*

			Photogrammetry, Remote Sensing and Spatial Information Sciences. vol. XLII-4/W2, International Society for Photogrammetry and Remote Sensing. pp. 117-120 . (doi:10.5194/isprs-archives-XLII-4-W2-117-2017).
Travel time to the nearest health facility (potentially providing routine immunization services)	Travel time to the nearest health facility providing routine immunization services in minutes	Continuous	[*Produced from locations of health facilities in Nigeria, excluding those locations that were reported not to be offering routine immunization services, using the methodology in*] Weiss, D.J. *et al*. (2018). A global map of travel time to cities to access inequalities in accessibility in 2015. *Nature*.

Table 2: Detailed description of DHS-derived covariates considered in the analysis

**Covariate**	**Description**	**Source**
Household wealth	Proportion of households whose wealth quintiles were classified as middle/richer/richest	NDHS 2018<br><br>National Population Commission - NPC and ICF, Nigeria Demographic and Health Survey 2018 - Final Report, Abuja, Nigeria: NPC and ICF. Available at http://dhsprogram.com/pubs/pdf/FR359/FR359.pdf, 2019
Maternal education	Proportion of mothers who had at least a primary education	NDHS 2018
Access to media	Proportion of mothers who had access to newspaper/radio/television at least once a week	NDHS 2018
Religion	Proportion of mothers who practised Islamic religion	NDHS 2018
Skilled birth attendance	Proportion of live births in the 5 years preceding the survey that were assisted by a skilled provider (i.e. doctor/nurse/auxiliary nurse/midwide)	NDHS 2018
Urban-rural	Urban and rural areas produced using WorldPop 2018 population	NDHS 2018, WorldPop 2018

	estimates and information obtained from the 2018 NDHS as described in the manuscript	Tatem AJ. WorldPop, open data for spatial demography. *Sci Data*. 2017; **4**:170004.

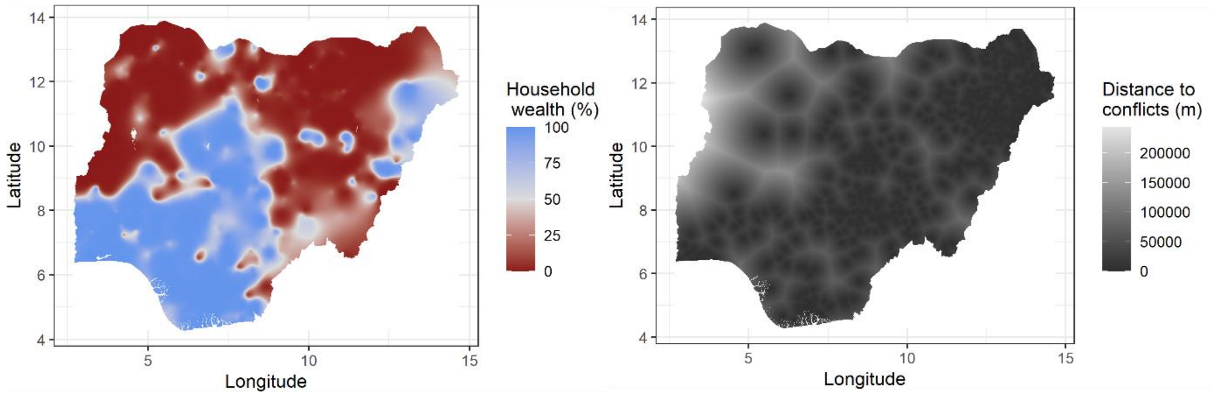

Figure 3: Maps of the remaining covariates selected for the study

Additional information on the INLA-SPDE approach and model fitting

The SPDE approach involves a triangulation of the spatial domain in order to approximate $\boldsymbol{\omega}$. A mesh was constructed for this approximation using the survey cluster locations and the boundary points of Nigeria. The maximum triangle edge length was set to be 0.05 degrees in the inner mesh (which is smaller than $r_0 \approx 0.48$ degrees) and 0.6 degrees in the outer mesh. The choice of these edge lengths was guided by the need to maintain a balance between the accuracy of the approximation and computational costs.

From each of the fitted models, we generated 1000 samples from the posterior distributions of the parameters of the model, as well as from the posterior predictive distributions of the modelled indicators for each of the prediction locations, i.e. the 1 x 1 km grid cells. The latter were then used to calculate the estimates of the remaining target indicators for each approach at the grid level using the formulae provided in the manuscript.

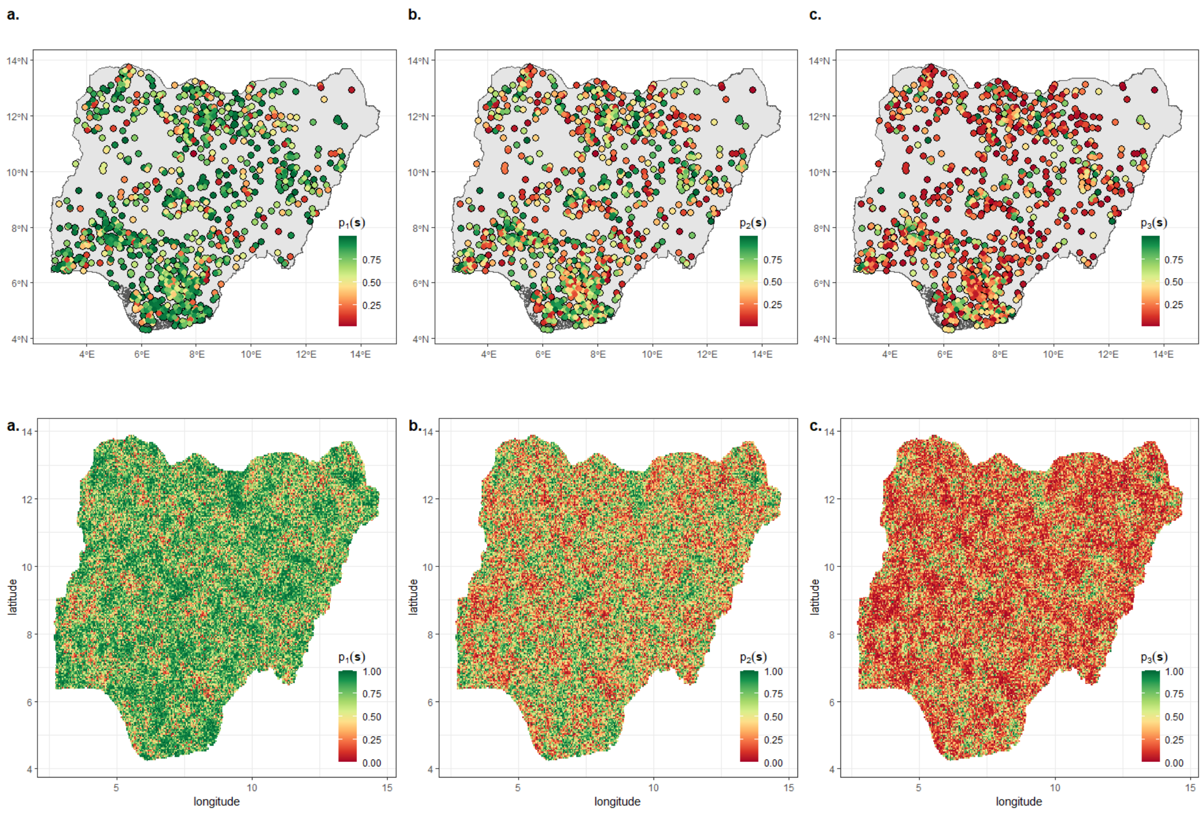

Figure 4: Spatially correlated point (top row) and grid (bottom row) level data for $p_1(\boldsymbol{s})$ (a, d), $p_2(\boldsymbol{s})$ (b, e) and $p_3(\boldsymbol{s})$ (c, f) used in the simulation study.

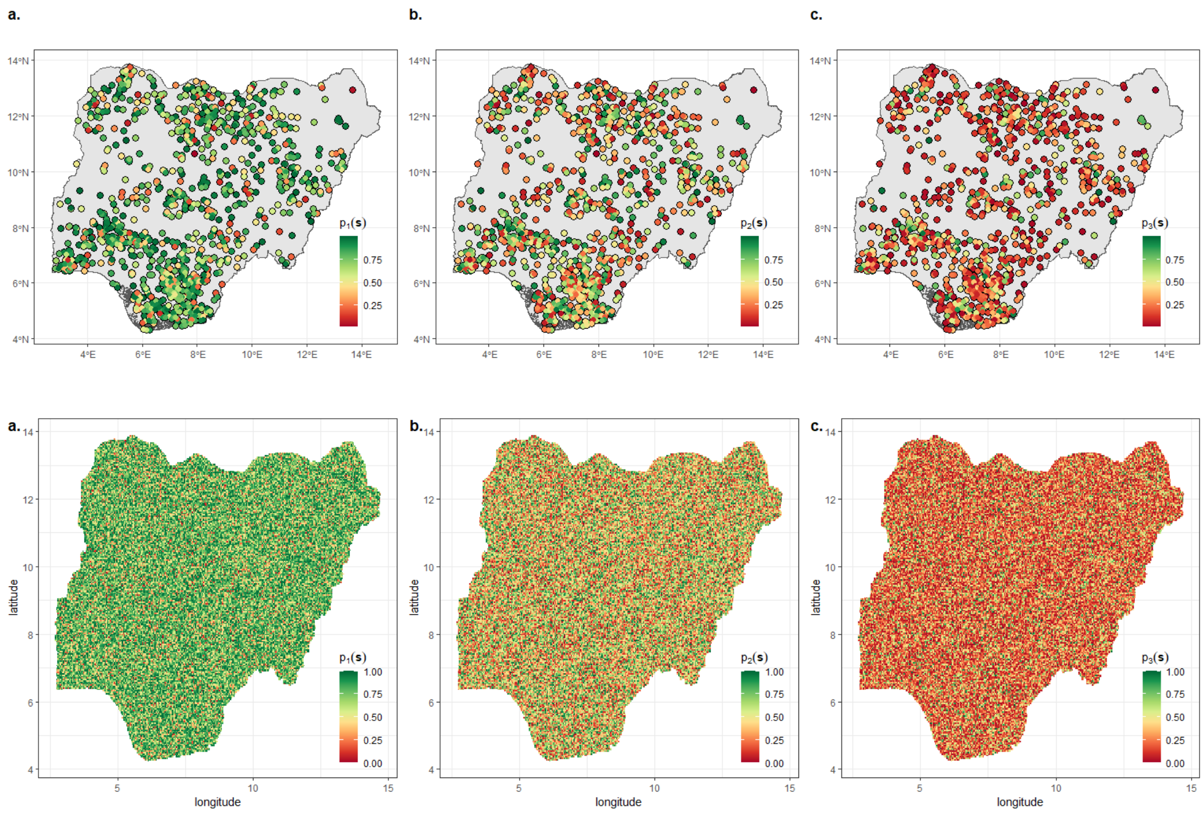

Figure 5: Spatially independent point (top row) and grid (bottom row) level data for $p_1(\boldsymbol{s})$ (a, d), $p_2(\boldsymbol{s})$ (b, e) and $p_3(\boldsymbol{s})$ (c, f) used in the simulation study.

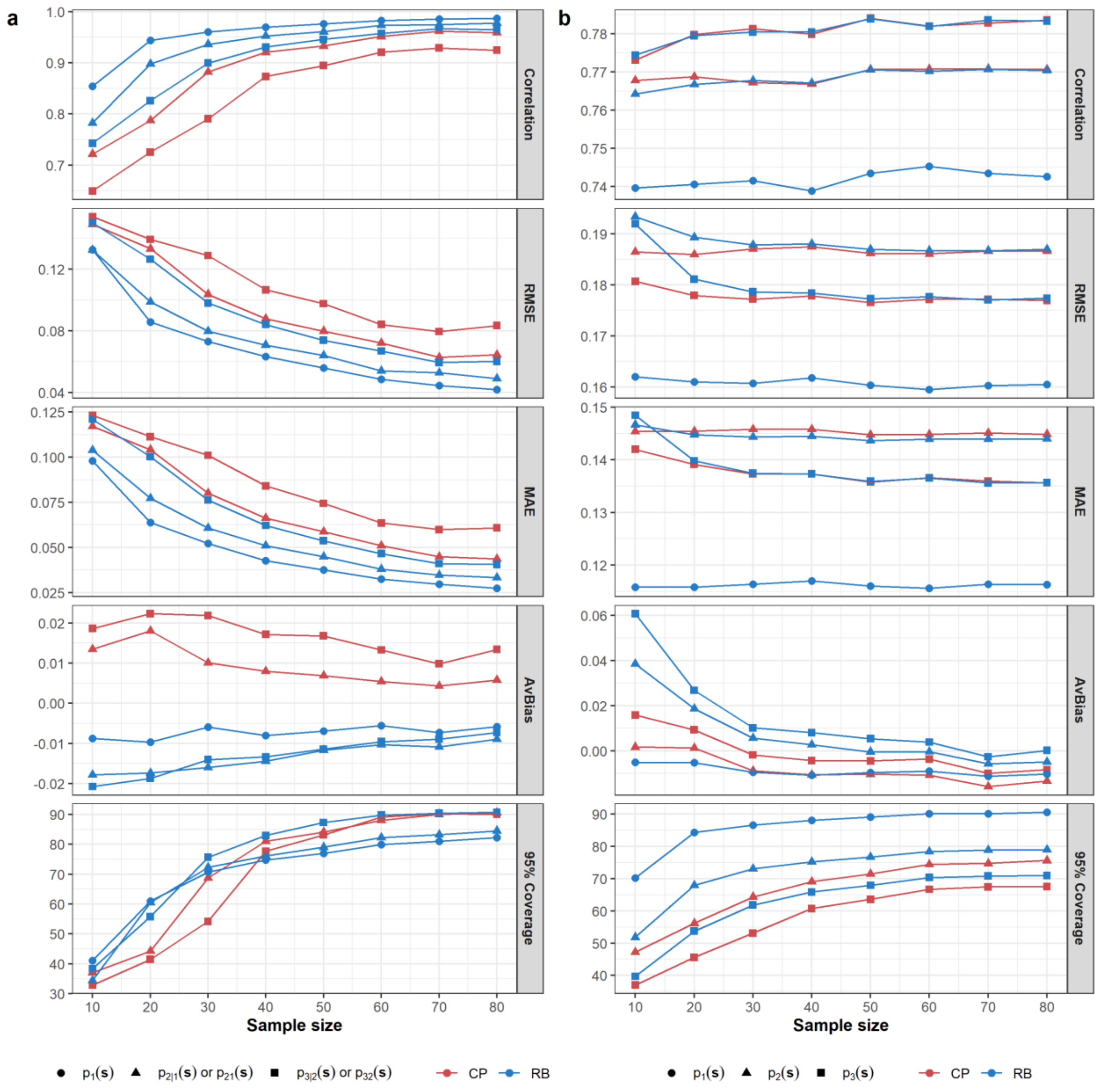

Figure 6: Predictive performance of the Conditional probability (CP) and ratio-based (RB) approaches based on different sample size distributions for spatially-correlated point-level data: (a) in-sample predictive performance based on the modelled indicators under each approach; (b) out-of-sample predictive performance for the target indicators based on a 5 × 5 km grid

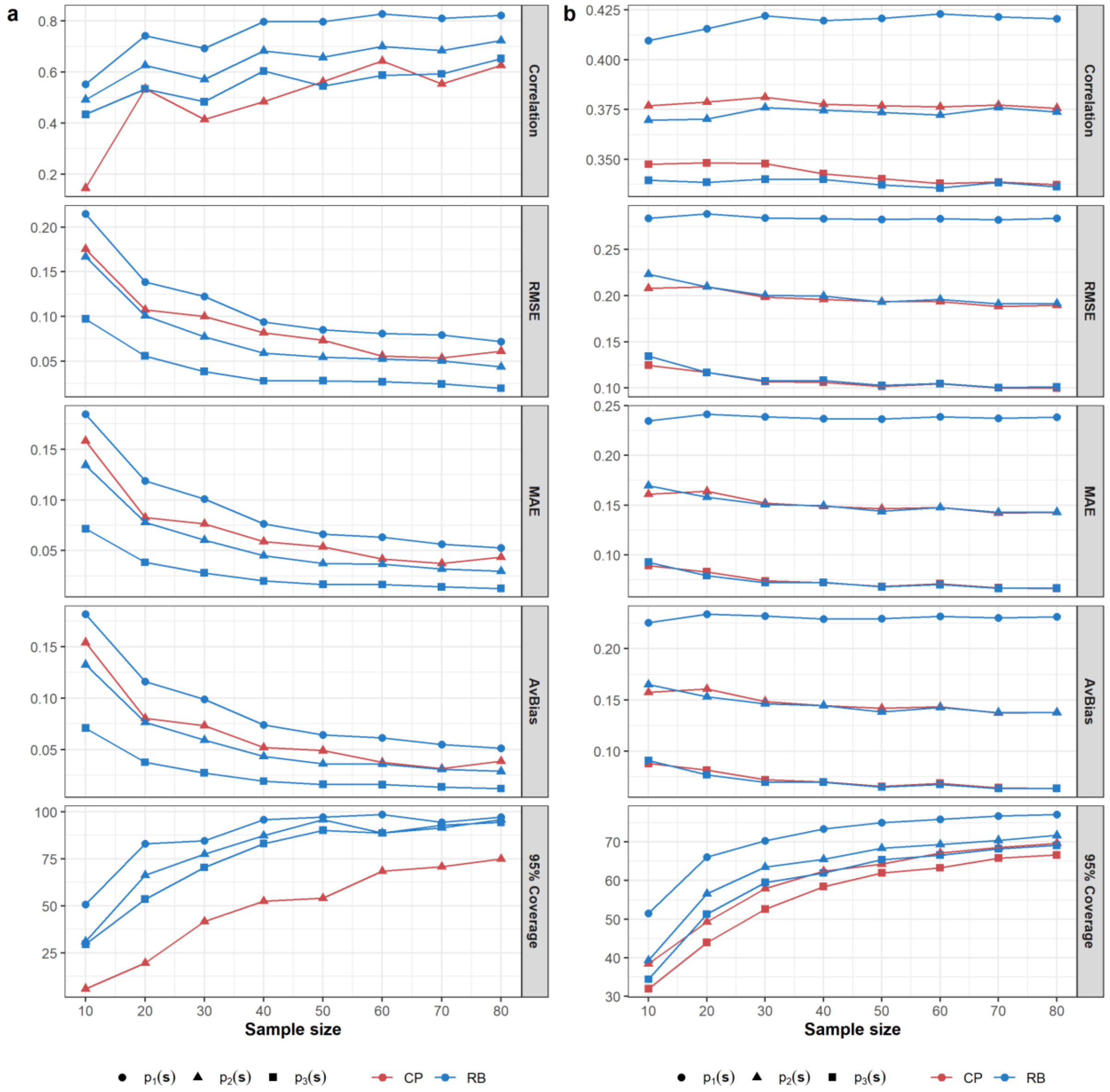

Figure 7: Predictive performance of the conditional probability (CP) and ratio-based (RB) approaches based on different sample size distributions for spatially-correlated data when $p_1(\boldsymbol{s}) \leq 0.3$ (i.e., the validation metrics were calculated using the observations and predictions for locations where $p_1(\boldsymbol{s}) \leq 0.3$ only): (a) in-sample prediction of the target indicators; (b) out-of-sample prediction of the target indicators over a 5×5 km grid. Note that $p_3(\boldsymbol{s})$ is not included in the in-sample predictions for the CP approach due to very small values of $p_1(\boldsymbol{s})$ resulting in non-receipt of the 2nd dose and hence zero sample size for $p_{3|2}(\boldsymbol{s})$, which is needed to calculate $p_3(\boldsymbol{s})$ for these cases.

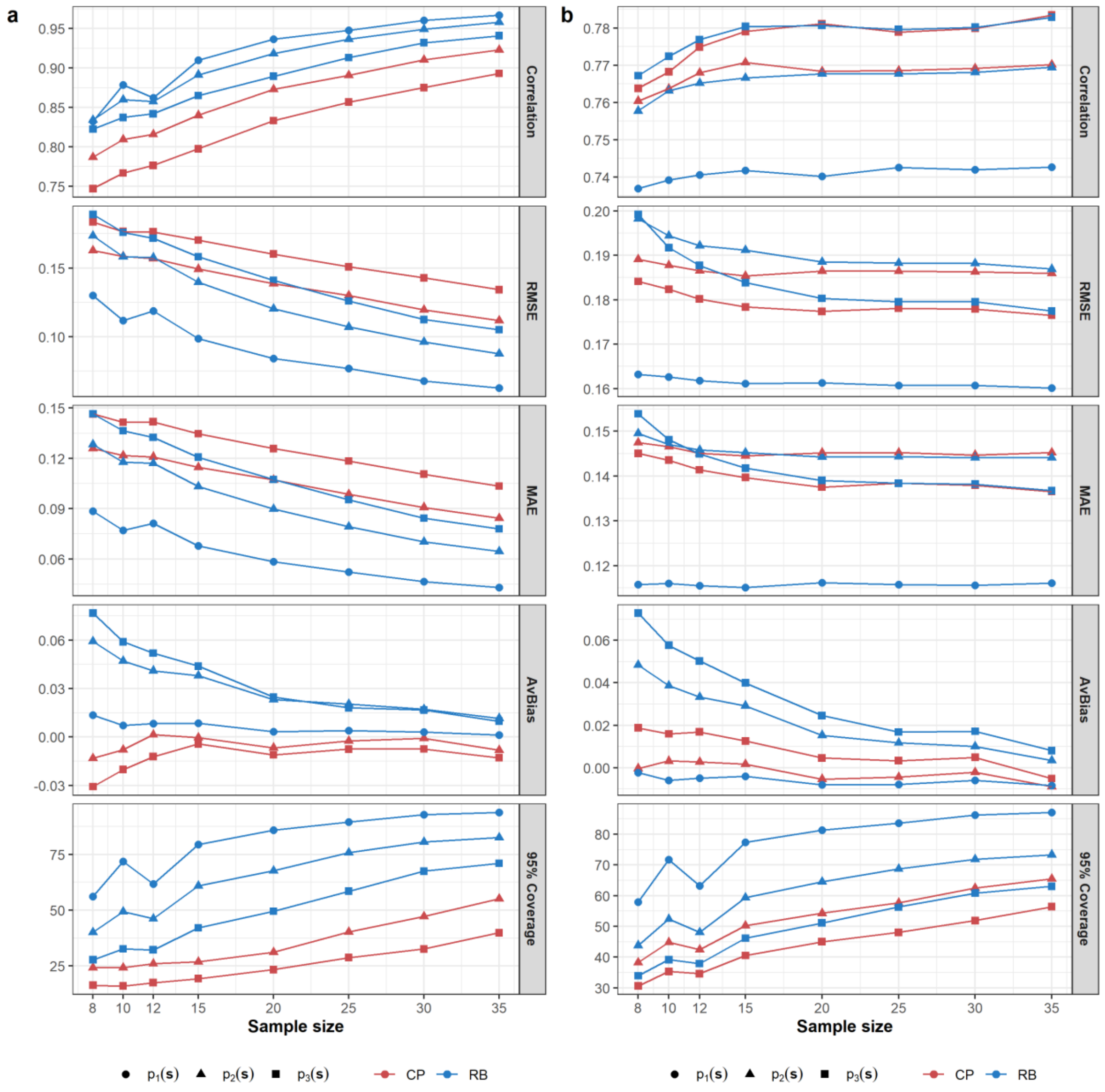

Figure 8: Predictive performance of the conditional probability (CP) and ratio-based (RB) approaches based on additional sample size distributions for spatially-correlated point-level data: (a) in-sample prediction of the target indicators; (b) out-of-sample prediction of the target indicators over a 5 × 5 km grid.

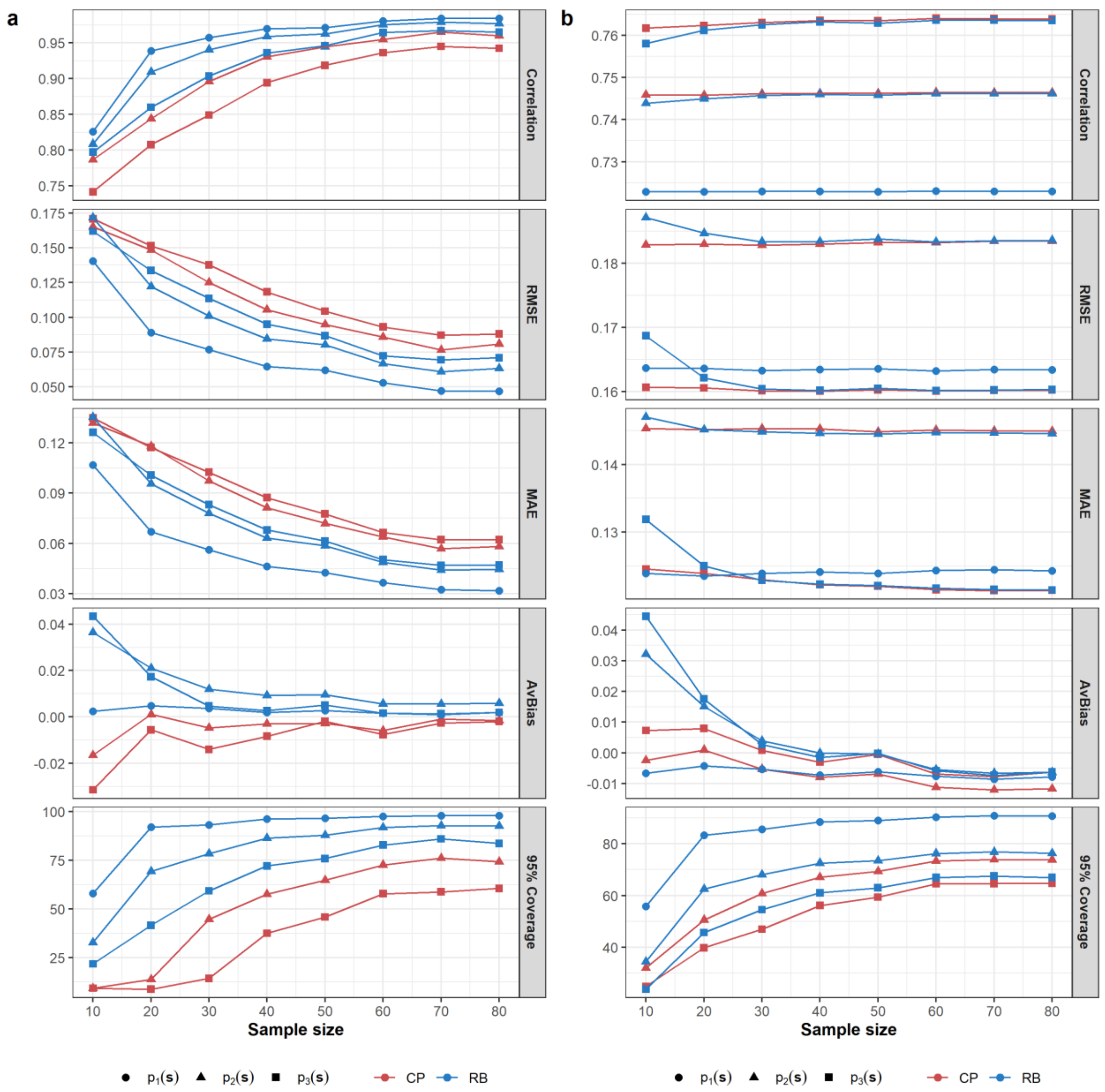

Figure 9: Predictive performance of the Conditional probability (CP) and ratio-based (RB) approaches based on different sample size distributions for spatially independent point-level data: (a) In-sample prediction of the target indicators; (b) out-of-sample prediction of the target indicators over a 5 × 5 km grid.

Table 3: Parameter estimates for the conditional probability (CP) approach

Parameter	Mean	Std. dev.	2.5%	97.5%
$p_1(\boldsymbol{s})$				
Intercept	-0.5146	0.3743	-1.2351	0.2432
Maternal education	1.5544	0.3240	0.9188	2.1911
Religion	-0.6269	0.2287	-1.0796	-0.1812
Skilled birth attendance	1.6682	0.2980	1.0887	2.2582
log(Livestock (pigs) density)	-0.0089	0.0446	-0.0972	0.0781
Urbanicity	0.0948	0.1425	-0.1852	0.3745
log(Night-time lights)	0.0104	0.0521	-0.0917	0.1128
Household wealth	0.3808	0.2395	-0.0891	0.8512
Proximity to national borders	0.0000	0.0016	-0.0034	0.0030
$\hat{r}$*	2.1576	0.7236	1.0115	3.8193
$\hat{\sigma}^2$	0.7806	0.2742	0.3543	1.4179
$\hat{\sigma}_\epsilon^2$	0.5587	0.1068	0.3668	0.7835
$p_{2\|1}(\boldsymbol{s})$				
Intercept	1.5371	0.4004	0.7602	2.3336
Maternal education	1.1100	0.4213	0.2858	1.9400
Religion	-0.2738	0.2737	-0.8129	0.2621
Skilled birth attendance	0.2100	0.3701	-0.5188	0.9345
log(Livestock (pigs) density)	-0.0444	0.0531	-0.1494	0.0591
Urbanicity	0.1929	0.1880	-0.1754	0.5625
log(Night-time lights)	-0.0452	0.0673	-0.1773	0.0868
Household wealth	0.1063	0.3230	-0.5266	0.7416
Proximity to national borders	-0.0009	0.0013	-0.0036	0.0017
$\hat{r}$*	1.0387	0.2998	0.5785	1.7476
$\hat{\sigma}^2$	0.6602	0.1897	0.3550	1.0940
$\hat{\sigma}_\epsilon^2$	0.2374	0.1430	0.0500	0.5905
$p_{3\|2}(\boldsymbol{s})$				
Intercept	0.9411	0.4258	0.0976	1.7736
Maternal education	0.4556	0.4279	-0.3848	1.2959
Religion	-0.0505	0.2831	-0.6051	0.5069
Skilled birth attendance	$p_1(\boldsymbol{s})$	$p_1(\boldsymbol{s})$	$p_1(\boldsymbol{s})$	$p_1(\boldsymbol{s})$
log(Livestock (pigs) density)	0.0185	0.0528	-0.0855	0.1218
Urbanicity	0.3640	0.1873	-0.0028	0.7325
log(Night-time lights)	0.0485	0.0676	-0.0837	0.1817
Household wealth	-0.0559	0.3207	-0.6866	0.5731
Proximity to national borders	0.0016	0.0014	-0.0014	0.0044
$\hat{r}$*	1.7013	0.8334	0.6107	3.8164
$\hat{\sigma}^2$	0.4650	0.1712	0.2069	0.8709
$\hat{\sigma}_\epsilon^2$	0.4564	0.1815	0.1897	0.8921

*The estimated ranges are 239 km, 115 km and 189 km respectively.

Table 4: Parameter estimates for ratio-based (RB) approach

$p_1(\boldsymbol{s})$				
**Parameter**	**Mean**	**Std. dev.**	**2.5%**	**97.5%**
Intercept	-0.5559	0.4429	-1.4291	0.3129
Maternal education	1.9974	0.2729	1.4627	2.5343
Distance to cultivated areas	0.0205	0.0266	-0.0316	0.0730
Skilled birth attendance	1.7951	0.2859	1.2391	2.3608
log(Travel time to urban areas)	0.0079	0.0394	-0.0695	0.0853
Urbanicity	0.0602	0.1447	-0.2237	0.3445
log(Distance to conflicts)	-0.0918	0.0696	-0.2289	0.0445
log(Livestock (pigs) density)	0.0238	0.0463	-0.0677	0.1143
Proximity to national borders	0.0001	0.0014	-0.0028	0.0028
$\hat{r}$*	1.5573	0.4515	0.8777	2.6365
$\hat{\sigma}^2$	0.6264	0.1654	0.3606	1.0061
$\hat{\sigma}_\epsilon^2$	0.5120	0.1105	0.3276	0.7599
$p_{21}(\boldsymbol{s})$				
Intercept	2.0541	0.6855	0.7165	3.4110
Maternal education	1.5277	0.4617	0.6229	2.4369
Distance to cultivated areas	-0.0566	0.0518	-0.1589	0.0448
Skilled birth attendance	0.7358	0.4586	-0.1618	1.6393
log(Travel time to urban areas)	0.0136	0.0679	-0.1199	0.1468
Urbanicity	0.2393	0.2503	-0.2497	0.7330
log(Distance to conflicts)	-0.0439	0.1116	-0.2617	0.1768
log(Livestock (pigs) density)	-0.0641	0.0720	-0.2061	0.0767
Proximity to national borders	-0.0020	0.0017	-0.0055	0.0013
$\hat{r}$*	1.0050	0.3571	0.5330	1.9105
$\hat{\sigma}^2$	1.2720	0.3822	0.6864	2.1758
$\hat{\sigma}_\epsilon^2$	2.3739	0.4726	1.6505	3.4914
$p_{32}(\boldsymbol{s})$				
Intercept	2.5474	0.8081	0.9807	4.1585
Maternal education	0.3257	0.5419	-0.7430	1.3866
Distance to cultivated areas	0.0973	0.0585	-0.0173	0.2127
Skilled birth attendance	1.7827	0.5293	0.7569	2.8351
log(Travel time to urban areas)	-0.0996	0.0775	-0.2530	0.0516
Urbanicity	0.3279	0.2943	-0.2483	0.9074
log(Distance to conflicts)	-0.2268	0.1250	-0.4750	0.0164
log(Livestock (pigs) density)	0.2052	0.0836	0.0417	0.3701
Proximity to national borders	0.0013	0.0022	-0.0032	0.0057
$\hat{r}$*	2.0292	0.9576	0.8208	4.4985
$\hat{\sigma}^2$	1.1411	0.4515	0.4849	2.2342
$\hat{\sigma}_\epsilon^2$	4.4402	0.5586	3.4303	5.6202

* The estimated ranges are 172 km, 111 km and 224 km respectively.

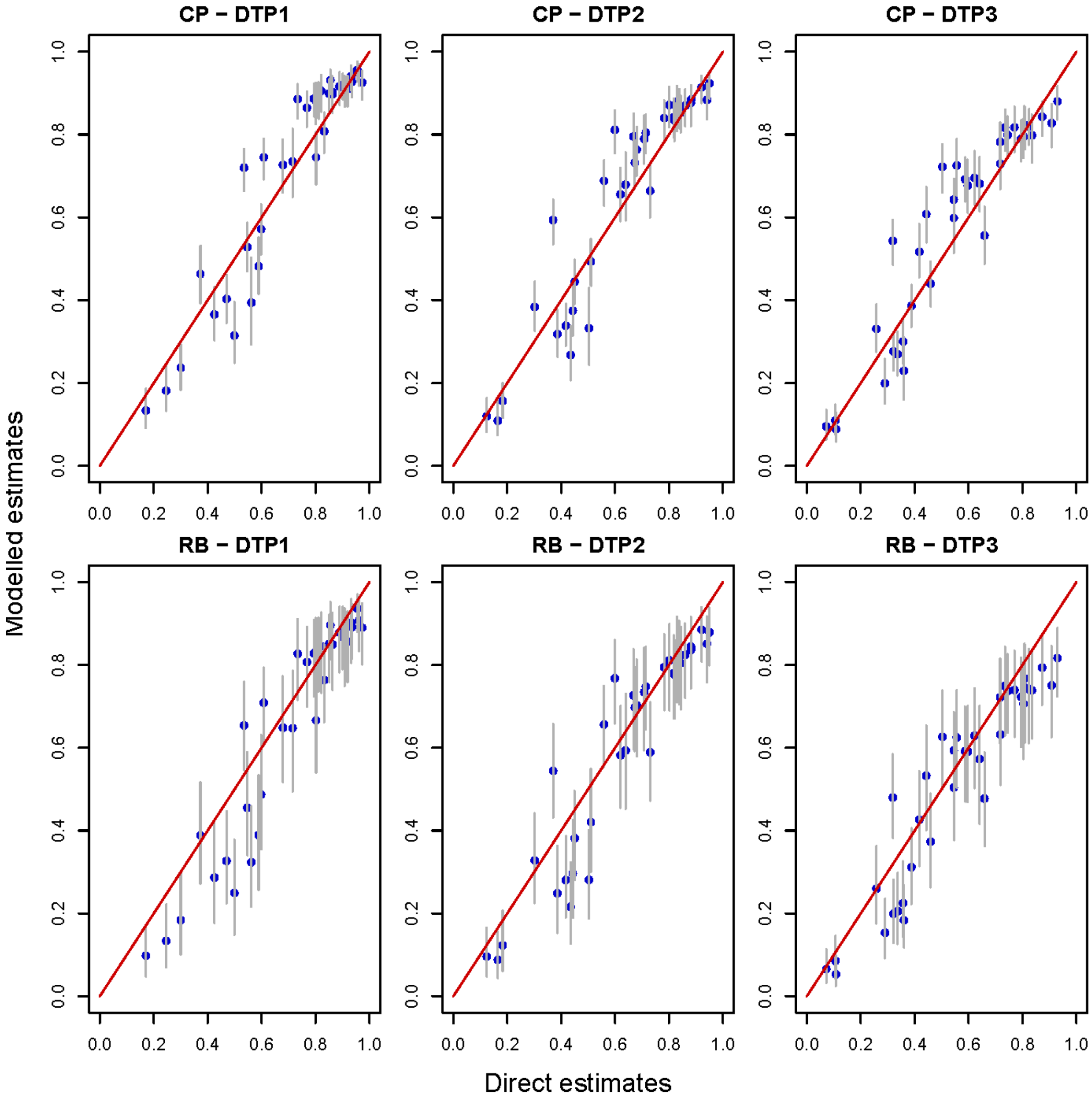

Figure 10: Model validation at the state level - direct survey estimates versus modelled estimates obtained using both the conditional probability (CP) and ratio-based (RB) approaches. The vertical grey lines are the 95% credible intervals of the modelled estimates.

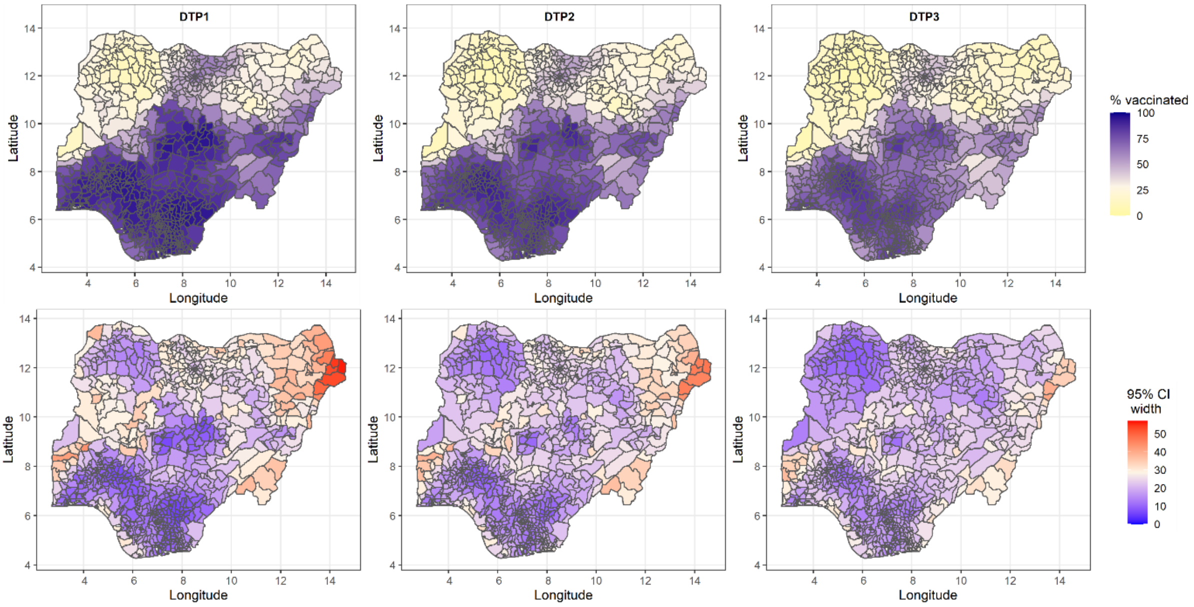

Figure 11: District-level estimates of DTP1-3 coverage and associated uncertainties for children aged 12-23 months.

Table 5: District-level estimates of DTP1-3 coverage and corresponding zero-dose estimates

District/LGA	State	Zero-dose estimate	Zero-dose estimate CI width	DTP1 coverage	DTP1 coverage 95% CI width	DTP2 coverage	DTP2 coverage 95% CI width	DTP3 coverage	DTP3 95% CI width
ABA NORTH	ABIA	1060	1644	0.94	0.09	0.91	0.11	0.84	0.15
ABA SOUTH	ABIA	3891	5301	0.94	0.08	0.91	0.11	0.85	0.13
ABADAM	BORNO	17948	9256	0.25	0.39	0.20	0.32	0.13	0.25
ABAJI	FCT	4744	4549	0.85	0.15	0.76	0.17	0.68	0.18
ABAK	AKWA IBOM	2142	2741	0.92	0.10	0.82	0.17	0.74	0.19
ABAKALIKI	EBONYI	1768	2695	0.95	0.07	0.92	0.10	0.81	0.14
ABEOKUTA NORTH	OGUN	5639	7080	0.86	0.18	0.75	0.23	0.66	0.24
ABEOKUTA SOUTH	OGUN	4546	6203	0.88	0.16	0.80	0.24	0.72	0.24
ABI	CROSS RIVER	1055	1527	0.96	0.06	0.91	0.11	0.78	0.18
ABOH-MBAISE	IMO	2792	3611	0.92	0.10	0.88	0.12	0.80	0.14
ABUA/ODUAL	RIVERS	8787	8604	0.83	0.16	0.79	0.18	0.72	0.18
ADAVI	KOGI	5185	6735	0.90	0.13	0.80	0.19	0.74	0.20
ADO	BENUE	2616	3741	0.94	0.09	0.89	0.12	0.80	0.17
ADO-EKITI	EKITI	2207	2939	0.95	0.06	0.93	0.08	0.89	0.10
ADO ODO/OTA	OGUN	15470	14611	0.89	0.11	0.83	0.13	0.76	0.14
AFIJIO	OYO	2026	2927	0.90	0.14	0.84	0.17	0.66	0.23
AFIKPO NORTH	EBONYI	1181	1542	0.96	0.05	0.91	0.10	0.82	0.13
AFIKPO SOUTH	EBONYI	1132	1518	0.96	0.05	0.91	0.10	0.83	0.13
AGAIE	NIGER	11428	9688	0.59	0.35	0.51	0.35	0.43	0.31
AGATU	BENUE	2980	4725	0.86	0.21	0.79	0.25	0.71	0.27
AGEGE	LAGOS	2337	3580	0.92	0.12	0.88	0.15	0.83	0.18

AGUATA	ANAMBRA	4115	4369	0.93	0.08	0.87	0.10	0.84	0.10
AGWARA	NIGER	6503	3411	0.32	0.36	0.23	0.28	0.14	0.19
AHIAZU-MBAISE	IMO	2064	2476	0.93	0.09	0.88	0.12	0.81	0.14
AHOADA EAST	RIVERS	4738	5197	0.85	0.16	0.81	0.17	0.74	0.18
AHOADA WEST	RIVERS	6189	6201	0.85	0.15	0.80	0.16	0.73	0.18
AIYEDADE	OSUN	2983	4093	0.91	0.12	0.85	0.15	0.74	0.18
AIYEDIRE	OSUN	1457	2119	0.92	0.12	0.86	0.16	0.74	0.21
AJAOKUTA	KOGI	3851	5258	0.87	0.18	0.76	0.25	0.69	0.25
AJEROMI/IFELODUN	LAGOS	3879	6241	0.94	0.10	0.90	0.12	0.85	0.17
AJINGI	KANO	17638	10009	0.51	0.28	0.42	0.25	0.36	0.23
AKAMKPA	CROSS RIVER	3849	5125	0.84	0.22	0.76	0.22	0.58	0.26
AKINYELE	OYO	3586	4835	0.93	0.10	0.84	0.16	0.68	0.21
AKKO	GOMBE	22525	10785	0.59	0.19	0.49	0.19	0.43	0.18
AKOKO-EDO	EDO	4903	5946	0.93	0.09	0.86	0.14	0.80	0.15
AKOKO NORTH EAST	ONDO	1429	2162	0.95	0.08	0.91	0.11	0.86	0.13
AKOKO NORTH WEST	ONDO	1873	2761	0.95	0.07	0.91	0.11	0.86	0.13
AKOKO SOUTH EAST	ONDO	728	1041	0.95	0.08	0.90	0.12	0.85	0.13
AKOKO SOUTH WEST	ONDO	2022	2956	0.95	0.07	0.92	0.10	0.87	0.12
AKPABUYO	CROSS RIVER	5135	7482	0.85	0.21	0.76	0.25	0.60	0.28
AKUKU TORU	RIVERS	5540	7040	0.87	0.16	0.82	0.18	0.76	0.19
AKURE NORTH	ONDO	1082	1687	0.95	0.08	0.92	0.10	0.87	0.12
AKURE SOUTH	ONDO	3259	5103	0.95	0.08	0.92	0.10	0.88	0.11
AKWANGA	NASARAWA	1430	2267	0.94	0.09	0.81	0.22	0.72	0.24
ALBASU	KANO	22178	9792	0.41	0.26	0.34	0.24	0.29	0.21
ALEIRO	KEBBI	10897	2729	0.20	0.20	0.13	0.14	0.09	0.12
ALIMOSHO	LAGOS	21340	21203	0.92	0.08	0.88	0.10	0.82	0.12
ALKALERI	BAUCHI	30121	18553	0.59	0.25	0.51	0.24	0.43	0.22

AMUWO ODOFIN	LAGOS	4028	5579	0.93	0.09	0.90	0.11	0.85	0.15
ANAMBRA EAST	ANAMBRA	1102	1286	0.92	0.09	0.88	0.12	0.85	0.13
ANAMBRA WEST	ANAMBRA	3694	4149	0.90	0.11	0.85	0.13	0.81	0.14
ANAOCHA	ANAMBRA	2631	2763	0.93	0.07	0.89	0.10	0.86	0.10
ANDONI	RIVERS	674	1119	0.93	0.12	0.88	0.16	0.82	0.18
ANINRI	ENUGU	987	1349	0.96	0.05	0.90	0.11	0.83	0.13
ANIOCHA NORTH	DELTA	1237	1846	0.91	0.13	0.87	0.15	0.83	0.16
ANIOCHA SOUTH	DELTA	2151	2986	0.91	0.12	0.88	0.14	0.83	0.15
ANKA	ZAMFARA	36408	6018	0.11	0.15	0.08	0.11	0.06	0.09
ANKPA	KOGI	7581	10747	0.86	0.20	0.80	0.23	0.73	0.24
APA	BENUE	3234	4536	0.86	0.20	0.80	0.22	0.72	0.23
APAPA*	LAGOS	NA	NA	NA	NA	NA	NA	NA	NA
ARDO-KOLA	TARABA	3210	3464	0.77	0.25	0.69	0.27	0.53	0.28
AREWA DANDI	KEBBI	24593	9723	0.29	0.28	0.22	0.22	0.16	0.17
ARGUNGU	KEBBI	31938	9228	0.25	0.22	0.19	0.19	0.14	0.15
AROCHUKWU	ABIA	2392	3023	0.93	0.09	0.86	0.14	0.73	0.17
ASA	KWARA	2629	3111	0.86	0.17	0.80	0.19	0.75	0.19
ASARI-TORU	RIVERS	3173	4474	0.90	0.15	0.86	0.16	0.81	0.17
ASKIRA/UBA	BORNO	8108	6527	0.66	0.28	0.60	0.27	0.49	0.26
ATAKUNMOSA EAST	OSUN	244	376	0.94	0.10	0.91	0.11	0.83	0.15
ATAKUNMOSA WEST	OSUN	1134	1477	0.93	0.09	0.90	0.11	0.82	0.14
ATIBA	OYO	6022	7655	0.82	0.23	0.77	0.24	0.63	0.27
ATISBO	OYO	20721	15992	0.46	0.42	0.41	0.39	0.32	0.33
AUGIE	KEBBI	11959	3353	0.21	0.22	0.16	0.18	0.12	0.13
AUYO	JIGAWA	21364	13550	0.51	0.31	0.37	0.30	0.28	0.25
AWE	NASARAWA	5197	5793	0.80	0.22	0.71	0.25	0.58	0.25
AWGU	ENUGU	1316	1814	0.96	0.06	0.88	0.14	0.83	0.14
AWKA NORTH	ANAMBRA	1014	1276	0.93	0.09	0.88	0.12	0.84	0.13
AWKA SOUTH	ANAMBRA	1495	1815	0.94	0.08	0.89	0.11	0.86	0.12

AYAMELUM	ANAMBRA	2428	3158	0.91	0.12	0.83	0.17	0.79	0.18
BABURA	JIGAWA	19309	13843	0.59	0.29	0.50	0.27	0.41	0.24
BADAGRY	LAGOS	5101	6404	0.86	0.17	0.80	0.21	0.74	0.22
BAGUDO	KEBBI	42818	15155	0.27	0.26	0.19	0.21	0.13	0.16
BAGWAI	KANO	22083	11467	0.46	0.28	0.40	0.26	0.35	0.22
BAKASSI	CROSS RIVER	111	192	0.83	0.30	0.74	0.33	0.58	0.36
BAKORI	KATSINA	22013	8752	0.39	0.24	0.36	0.22	0.32	0.21
BAKURA	ZAMFARA	26808	4377	0.11	0.15	0.08	0.11	0.06	0.09
BALANGA	GOMBE	20353	11785	0.53	0.27	0.47	0.25	0.39	0.23
BALI	TARABA	19904	14491	0.59	0.30	0.50	0.26	0.40	0.24
BAMA	BORNO	38486	36824	0.45	0.53	0.38	0.46	0.25	0.34
BARDE	YOBE	21089	8220	0.30	0.27	0.26	0.24	0.18	0.19
BARKIN LADI	PLATEAU	1611	2933	0.96	0.07	0.90	0.14	0.80	0.19
BARUTEN	KWARA	34451	8733	0.14	0.22	0.11	0.18	0.08	0.13
BASSA	KOGI	4244	6099	0.85	0.21	0.75	0.27	0.67	0.28
BASSA	PLATEAU	4422	5972	0.90	0.13	0.84	0.17	0.74	0.20
BATAGARAWA	KATSINA	30992	12332	0.33	0.27	0.28	0.24	0.24	0.22
BATSARI	KATSINA	33779	14357	0.28	0.31	0.25	0.28	0.20	0.23
BAUCHI	BAUCHI	52327	30809	0.49	0.30	0.43	0.29	0.35	0.25
BAURE	KATSINA	17481	13114	0.57	0.32	0.49	0.31	0.39	0.27
BAYO	BORNO	13978	5584	0.30	0.28	0.23	0.24	0.19	0.20
BEBEJI	KANO	24712	11877	0.44	0.27	0.32	0.23	0.28	0.21
BEKWARRA	CROSS RIVER	1505	2339	0.92	0.12	0.86	0.17	0.70	0.25
BENDE	ABIA	1688	2281	0.95	0.07	0.87	0.13	0.76	0.17
BIASE	CROSS RIVER	3383	4272	0.89	0.14	0.83	0.16	0.67	0.22
BICHI	KANO	35000	15935	0.45	0.25	0.38	0.24	0.33	0.21

BIDA	NIGER	15280	12281	0.53	0.38	0.45	0.36	0.40	0.34
BILLIRI	GOMBE	5937	6790	0.85	0.17	0.76	0.22	0.67	0.23
BINDAWA	KATSINA	22807	8132	0.31	0.25	0.26	0.22	0.23	0.19
BINJI	SOKOTO	16487	4971	0.19	0.24	0.13	0.19	0.10	0.15
BIRINIWA	JIGAWA	15994	8102	0.46	0.27	0.36	0.26	0.25	0.20
BIRNIN GWARI	KADUNA	45152	20218	0.29	0.32	0.23	0.27	0.20	0.23
BIRNIN KEBBI	KEBBI	46047	12720	0.26	0.21	0.17	0.16	0.13	0.14
BIRNIN KUDU	JIGAWA	48343	17200	0.35	0.23	0.28	0.20	0.22	0.17
BIRNIN MAGAJI/KIYAW	ZAMFARA	36172	7795	0.14	0.18	0.13	0.16	0.10	0.14
BIU	BORNO	27374	16077	0.39	0.36	0.31	0.31	0.25	0.26
BODINGA	SOKOTO	35291	7246	0.15	0.17	0.09	0.13	0.08	0.11
BOGORO	BAUCHI	4231	5146	0.81	0.23	0.76	0.24	0.66	0.26
BOKI	CROSS RIVER	1993	3577	0.94	0.11	0.87	0.17	0.70	0.27
BOKKOS	PLATEAU	2128	3722	0.95	0.09	0.86	0.18	0.75	0.21
BOLUWADURO	OSUN	1325	1731	0.91	0.12	0.88	0.13	0.83	0.15
BOMADI	DELTA	3081	3165	0.80	0.20	0.75	0.21	0.67	0.22
BONNY	RIVERS	931	1732	0.94	0.12	0.88	0.16	0.83	0.18
BORGU	NIGER	29524	12260	0.28	0.30	0.21	0.22	0.15	0.17
BORIPE	OSUN	2303	2846	0.91	0.12	0.88	0.13	0.82	0.15
BORSARI	YOBE	17705	7656	0.28	0.31	0.25	0.27	0.17	0.21
BOSSO	NIGER	9582	8594	0.71	0.26	0.60	0.26	0.54	0.25
BRASS	BAYELSA	6202	5431	0.72	0.25	0.67	0.24	0.59	0.23
BUJI	JIGAWA	14726	5143	0.31	0.24	0.24	0.20	0.19	0.17
BUKKUYUM	ZAMFARA	43983	7874	0.12	0.16	0.08	0.12	0.06	0.10
BUKURU	BENUE	7864	8144	0.81	0.19	0.75	0.20	0.61	0.22
BUNGUDU	ZAMFARA	70628	11738	0.12	0.15	0.11	0.13	0.09	0.11
BUNKURE	KANO	20860	11339	0.51	0.27	0.41	0.25	0.36	0.22
BUNZA	KEBBI	18450	5619	0.22	0.24	0.13	0.18	0.09	0.13

BURUTU	DELTA	8388	8584	0.76	0.24	0.71	0.24	0.62	0.25
BWARI	FCT	2475	3122	0.94	0.08	0.90	0.11	0.84	0.13
CALABAR MUNICIPAL	CROSS RIVER	3165	4444	0.90	0.14	0.81	0.19	0.68	0.23
CALABAR SOUTH	CROSS RIVER	1957	2705	0.90	0.14	0.80	0.20	0.67	0.24
CHANCHAGA	NIGER	9126	10038	0.77	0.25	0.65	0.29	0.60	0.28
CHARANCHI	KATSINA	21682	8988	0.32	0.28	0.28	0.25	0.24	0.23
CHIBOK	BORNO	8194	6906	0.52	0.40	0.46	0.38	0.36	0.32
CHIKUN	KADUNA	15502	14570	0.84	0.15	0.67	0.25	0.63	0.24
DALA	KANO	19741	18246	0.78	0.21	0.71	0.22	0.66	0.23
DAMATURU	YOBE	13198	7115	0.31	0.37	0.27	0.33	0.20	0.26
DAMBAN	BAUCHI	27559	10563	0.30	0.27	0.25	0.23	0.20	0.19
DAMBATTA	KANO	20085	11625	0.54	0.27	0.46	0.25	0.39	0.22
DAMBOA	BORNO	28072	18895	0.45	0.37	0.38	0.33	0.28	0.27
DAN MUSA	KATSINA	17860	7857	0.31	0.30	0.28	0.27	0.24	0.24
DANDI	KEBBI	21451	8819	0.27	0.30	0.18	0.23	0.12	0.17
DANDUME	KATSINA	22882	9598	0.30	0.29	0.26	0.27	0.23	0.24
DANGE-SHUNI	SOKOTO	52567	10017	0.15	0.16	0.09	0.11	0.07	0.10
DANJA	KATSINA	14256	7174	0.51	0.25	0.44	0.23	0.40	0.21
DARAZO	BAUCHI	58084	17783	0.25	0.23	0.20	0.20	0.16	0.16
DASS	BAUCHI	6258	6472	0.69	0.33	0.63	0.31	0.54	0.30
DAURA	KATSINA	28635	15007	0.46	0.28	0.40	0.27	0.33	0.24
DAWAKIN KUDU	KANO	22146	13218	0.60	0.24	0.52	0.23	0.46	0.23
DAWAKIN TOFA	KANO	28758	14738	0.58	0.22	0.52	0.21	0.45	0.20
DEGEMA	RIVERS	4649	6087	0.91	0.12	0.86	0.14	0.81	0.16
DEKINA	KOGI	9024	11392	0.83	0.21	0.74	0.25	0.67	0.24
DEMSA	ADAMAWA	1968	2395	0.87	0.16	0.79	0.19	0.68	0.22
DIKWA	BORNO	14889	12616	0.38	0.53	0.31	0.47	0.20	0.34

DOGUWA	KANO	17362	9522	0.48	0.29	0.36	0.25	0.29	0.21
DOMA	NASARAWA	3841	4978	0.88	0.16	0.76	0.21	0.68	0.22
DONGA	TARABA	8922	9444	0.72	0.30	0.67	0.30	0.52	0.30
DUKKU	GOMBE	40438	9473	0.19	0.19	0.15	0.16	0.12	0.13
DUNUKOFIA	ANAMBRA	965	1083	0.93	0.08	0.90	0.10	0.87	0.11
DUTSE	JIGAWA	37712	15600	0.40	0.25	0.32	0.22	0.27	0.19
DUTSI	KATSINA	16696	7200	0.38	0.27	0.33	0.23	0.27	0.21
DUTSIN MA	KATSINA	24553	11869	0.34	0.32	0.30	0.29	0.26	0.26
EASTERN OBOLO	AKWA IBOM	911	1355	0.90	0.14	0.79	0.22	0.71	0.24
EBONYI	EBONYI	1123	1692	0.95	0.08	0.91	0.11	0.81	0.15
EDATI	NIGER	32827	19403	0.50	0.30	0.42	0.26	0.35	0.24
EDE NORTH	OSUN	1391	1931	0.92	0.12	0.88	0.14	0.81	0.17
EDE SOUTH	OSUN	1200	1685	0.92	0.11	0.89	0.13	0.80	0.18
EDU	KWARA	12779	11966	0.71	0.27	0.60	0.26	0.54	0.25
EFON	EKITI	1012	1405	0.94	0.08	0.92	0.10	0.86	0.12
EGBADO NORTH	OGUN	7070	9008	0.81	0.24	0.69	0.30	0.58	0.30
EGBADO SOUTH	OGUN	5949	7091	0.82	0.21	0.73	0.25	0.64	0.26
EGBEDA	OYO	3864	5089	0.93	0.10	0.82	0.17	0.69	0.21
EGBEDORE	OSUN	2266	2902	0.90	0.12	0.87	0.15	0.79	0.17
EGOR	EDO	3327	5758	0.93	0.12	0.87	0.16	0.84	0.16
EHIME -MBANO	IMO	1984	2264	0.92	0.09	0.86	0.13	0.78	0.15
EJIGBO	OSUN	2275	3104	0.90	0.14	0.85	0.18	0.74	0.21
EKEREMOR	BAYELSA	16098	15758	0.69	0.30	0.63	0.29	0.54	0.28
EKET	AKWA IBOM	2701	3834	0.91	0.13	0.73	0.29	0.66	0.28
EKITI	KWARA	964	1449	0.93	0.10	0.90	0.13	0.86	0.14
EKITI EAST	EKITI	1351	2176	0.95	0.08	0.92	0.12	0.87	0.14
EKITI SOUTH-WEST	EKITI	1608	2316	0.95	0.08	0.92	0.09	0.87	0.11

EKITI WEST	EKITI	2133	2916	0.94	0.08	0.92	0.09	0.87	0.11
EKWUSIGO	ANAMBRA	2717	2848	0.92	0.09	0.88	0.11	0.85	0.11
ELEME	RIVERS	2607	3149	0.93	0.09	0.89	0.12	0.84	0.13
EMUOHA	RIVERS	5353	5722	0.86	0.15	0.82	0.16	0.76	0.17
EMURE	EKITI	768	1154	0.95	0.07	0.93	0.09	0.88	0.11
ENUGU EAST	ENUGU	2006	3210	0.96	0.07	0.90	0.11	0.86	0.13
ENUGU NORTH	ENUGU	865	1439	0.96	0.06	0.90	0.12	0.87	0.14
ENUGU SOUTH	ENUGU	1025	1687	0.96	0.06	0.90	0.13	0.86	0.14
EPE	LAGOS	2230	3693	0.92	0.12	0.84	0.20	0.77	0.22
ESAN NORTH-EAST	EDO	1132	1729	0.94	0.10	0.89	0.14	0.84	0.15
ESAN CENTRAL	EDO	1059	1640	0.94	0.09	0.90	0.14	0.85	0.16
ESAN SOUTH-EAST	EDO	2571	3334	0.91	0.11	0.86	0.15	0.81	0.16
ESAN WEST	EDO	1199	1869	0.94	0.09	0.90	0.14	0.85	0.15
ESE ODO	ONDO	5860	9002	0.80	0.30	0.76	0.31	0.71	0.30
ESIT EKET	AKWA IBOM	1564	2133	0.89	0.16	0.73	0.27	0.63	0.26
ESSIEN UDIM	AKWA IBOM	2388	3139	0.94	0.09	0.86	0.14	0.78	0.16
ETCHE	RIVERS	5565	6296	0.90	0.11	0.86	0.12	0.79	0.16
ETHIOPE EAST	DELTA	3332	4457	0.91	0.12	0.86	0.15	0.81	0.16
ETHIOPE WEST	DELTA	2791	4322	0.92	0.13	0.87	0.16	0.82	0.17
ETI OSA	LAGOS	1503	2339	0.94	0.09	0.89	0.14	0.84	0.15
ETIM EKPO	AKWA IBOM	1386	2011	0.94	0.09	0.86	0.14	0.79	0.16
ETINAN	AKWA IBOM	3181	3864	0.90	0.12	0.77	0.18	0.68	0.20
ETSAKO CENTRAL	EDO	1328	1813	0.92	0.11	0.84	0.17	0.78	0.19
ETSAKO EAST	EDO	2606	3426	0.91	0.12	0.82	0.19	0.76	0.20
ETSAKO WEST	EDO	2137	3199	0.94	0.10	0.88	0.15	0.83	0.16

ETUNG	CROSS RIVER	1203	2336	0.92	0.16	0.85	0.22	0.67	0.31
EWEKORO	OGUN	1742	1849	0.86	0.14	0.76	0.21	0.66	0.22
EZEAGU	ENUGU	1576	2164	0.94	0.08	0.87	0.13	0.83	0.14
EZINIHITTE	IMO	2173	2844	0.93	0.09	0.89	0.12	0.81	0.14
EZZA NORTH	EBONYI	1256	1866	0.95	0.07	0.92	0.10	0.84	0.13
EZZA SOUTH	EBONYI	1025	1480	0.96	0.06	0.92	0.08	0.83	0.12
FAGGE	KANO	9757	10097	0.78	0.23	0.71	0.24	0.66	0.26
FAKAI	KEBBI	23300	8365	0.22	0.28	0.14	0.20	0.09	0.15
FASKARI	KATSINA	34658	10198	0.23	0.23	0.20	0.20	0.17	0.18
FIKA	YOBE	28221	10383	0.32	0.25	0.27	0.22	0.21	0.18
FUFORE	ADAMAWA	16393	16118	0.74	0.26	0.64	0.26	0.51	0.25
FUNAKAYE	GOMBE	37564	10915	0.23	0.22	0.17	0.20	0.14	0.16
FUNE	YOBE	53891	21152	0.32	0.27	0.28	0.24	0.22	0.18
FUNTUA	KATSINA	30192	13297	0.37	0.28	0.33	0.26	0.30	0.23
GABASAWA	KANO	17546	11193	0.60	0.26	0.51	0.25	0.44	0.24
GADA	SOKOTO	29097	9794	0.22	0.26	0.15	0.21	0.12	0.17
GAGARAWA	JIGAWA	6791	5342	0.60	0.31	0.45	0.29	0.35	0.25
GAMAWA	BAUCHI	46262	16737	0.30	0.25	0.24	0.21	0.19	0.18
GANJUWA	BAUCHI	44580	16682	0.32	0.25	0.26	0.22	0.20	0.17
GANYE	ADAMAWA	6851	9005	0.79	0.28	0.69	0.29	0.58	0.29
GARKI	JIGAWA	12848	8607	0.63	0.25	0.53	0.24	0.44	0.22
GARKO	KANO	19397	9248	0.43	0.27	0.35	0.25	0.30	0.22
GARUM MALLAM	KANO	12322	6472	0.49	0.27	0.38	0.25	0.34	0.22
GASHAKA	TARABA	6885	6635	0.65	0.34	0.54	0.34	0.43	0.29
GASSOL	TARABA	25938	14254	0.50	0.28	0.42	0.25	0.32	0.21
GAYA	KANO	24453	11811	0.44	0.27	0.36	0.25	0.31	0.22
GBAKO	NIGER	15897	9204	0.51	0.29	0.44	0.27	0.37	0.25
GBOKO	BENUE	11288	13457	0.85	0.18	0.79	0.19	0.68	0.22

GBONYIN	EKITI	1208	1643	0.96	0.06	0.93	0.08	0.89	0.11
GEIDAM	YOBE	25767	12111	0.25	0.35	0.22	0.30	0.15	0.22
GEZAWA	KANO	16062	10608	0.65	0.23	0.57	0.24	0.51	0.22
GIADE	BAUCHI	33006	10449	0.25	0.24	0.19	0.21	0.15	0.17
GIRIE	ADAMAWA	5614	7149	0.86	0.18	0.76	0.22	0.64	0.26
GIWA	KADUNA	36206	19151	0.49	0.27	0.41	0.25	0.36	0.23
GOKANA	RIVERS	3510	4974	0.93	0.10	0.89	0.12	0.84	0.14
GOMBE	GOMBE	9424	6848	0.55	0.33	0.42	0.31	0.38	0.28
GOMBI	ADAMAWA	12053	10682	0.64	0.32	0.56	0.31	0.47	0.30
GORONYO	SOKOTO	36666	9002	0.18	0.20	0.12	0.16	0.09	0.13
GUBIO	BORNO	28020	11525	0.21	0.32	0.17	0.28	0.11	0.21
GUDU	SOKOTO	13239	6849	0.27	0.38	0.21	0.30	0.15	0.23
GUJBA	YOBE	19500	10947	0.33	0.38	0.27	0.32	0.20	0.24
GULANI	YOBE	19843	7711	0.28	0.28	0.22	0.23	0.17	0.19
GUMA	BENUE	8330	7900	0.81	0.18	0.72	0.22	0.61	0.23
GUMEL	JIGAWA	9374	9161	0.61	0.38	0.48	0.36	0.40	0.32
GUMMI	ZAMFARA	36842	7957	0.13	0.19	0.09	0.14	0.07	0.11
GURARA	NIGER	2821	3312	0.89	0.12	0.84	0.16	0.78	0.17
GURI	JIGAWA	15737	7696	0.39	0.30	0.30	0.26	0.22	0.21
GUSAU	ZAMFARA	64792	12378	0.15	0.16	0.13	0.15	0.11	0.12
GUYUK	ADAMAWA	11554	8018	0.59	0.28	0.52	0.26	0.43	0.24
GUZAMALA	BORNO	13252	6775	0.25	0.38	0.20	0.32	0.12	0.21
GWADABAWA	SOKOTO	39005	9118	0.19	0.19	0.11	0.14	0.09	0.12
GWAGWALADA	FCT	6308	7131	0.92	0.09	0.88	0.12	0.80	0.14
GWALE	KANO	17714	17682	0.78	0.22	0.72	0.23	0.67	0.23
GWANDU	KEBBI	28315	6447	0.17	0.19	0.12	0.14	0.09	0.11
GWARAM	JIGAWA	42527	14320	0.30	0.23	0.23	0.19	0.19	0.17
GWARZO	KANO	21912	13652	0.51	0.31	0.43	0.28	0.37	0.26
GWER EAST	BENUE	4515	5642	0.87	0.16	0.82	0.19	0.72	0.20

GWER WEST	BENUE	2755	4101	0.88	0.18	0.82	0.20	0.74	0.22
GWIWA	JIGAWA	10447	5396	0.48	0.27	0.40	0.24	0.34	0.21
GWOZA	BORNO	25479	28909	0.56	0.49	0.50	0.46	0.37	0.39
HADEJIA	JIGAWA	7305	6095	0.55	0.38	0.41	0.38	0.33	0.33
HAWUL	BORNO	15068	11045	0.49	0.38	0.41	0.34	0.33	0.29
HONG	ADAMAWA	7627	6829	0.70	0.27	0.63	0.26	0.52	0.26
IBADAN NORTH	OYO	3880	5915	0.93	0.10	0.84	0.18	0.69	0.23
IBADAN NORTH EAST	OYO	3337	5222	0.93	0.11	0.83	0.19	0.69	0.26
IBADAN NORTH WEST	OYO	2437	3932	0.93	0.11	0.84	0.18	0.70	0.25
IBADAN SOUTH EAST	OYO	1959	3100	0.93	0.11	0.82	0.20	0.69	0.26
IBADAN SOUTH WEST	OYO	3606	5605	0.93	0.11	0.83	0.19	0.70	0.23
IBAJI	KOGI	3135	3677	0.89	0.12	0.80	0.17	0.74	0.19
IBARAPA CENTRAL	OYO	2919	4366	0.84	0.24	0.75	0.28	0.64	0.31
IBARAPA EAST	OYO	3106	4608	0.86	0.21	0.79	0.24	0.64	0.29
IBARAPA NORTH	OYO	3560	5378	0.82	0.27	0.75	0.30	0.61	0.34
IBEJU LEKKI	LAGOS	1261	2239	0.93	0.13	0.84	0.23	0.78	0.23
IBENO	AKWA IBOM	1662	2467	0.89	0.16	0.73	0.28	0.65	0.27
IBESIKPO ASUTAN	AKWA IBOM	3016	3390	0.88	0.13	0.76	0.19	0.65	0.21
IBI	TARABA	5423	4631	0.69	0.26	0.63	0.27	0.49	0.24
IBIONO IBOM	AKWA IBOM	4081	4842	0.89	0.14	0.79	0.19	0.67	0.22
IDAH	KOGI	1085	1710	0.90	0.15	0.81	0.24	0.75	0.27
IDANRE	ONDO	1912	2841	0.92	0.12	0.89	0.13	0.83	0.15
IDEATO NORTH	IMO	2566	2645	0.91	0.09	0.84	0.13	0.79	0.13
IDEATO SOUTH	IMO	2161	2441	0.91	0.10	0.84	0.13	0.79	0.14
IDEMILI NORTH	ANAMBRA	5015	5304	0.92	0.08	0.89	0.10	0.87	0.11
IDEMILI SOUTH	ANAMBRA	4316	4279	0.92	0.08	0.89	0.10	0.86	0.11

IDO	OYO	2030	2557	0.92	0.10	0.82	0.16	0.68	0.21
IDO-OSI	EKITI	1294	1718	0.95	0.07	0.93	0.09	0.89	0.11
IFAKO/IJAYE	LAGOS	5238	6750	0.92	0.10	0.88	0.13	0.82	0.14
IFE CENTRAL	OSUN	483	737	0.93	0.10	0.90	0.13	0.82	0.17
IFE EAST	OSUN	3116	4814	0.93	0.11	0.90	0.13	0.83	0.16
IFE NORTH	OSUN	1657	2365	0.92	0.11	0.88	0.14	0.79	0.17
IFE SOUTH	OSUN	3331	5168	0.91	0.14	0.88	0.15	0.81	0.17
IFEDAYO	OSUN	396	551	0.94	0.09	0.91	0.10	0.86	0.14
IFEDORE	ONDO	1924	2882	0.95	0.08	0.92	0.10	0.88	0.12
IFELODUN	KWARA	7433	7005	0.84	0.15	0.78	0.17	0.73	0.18
IFELODUN	OSUN	1467	1901	0.90	0.13	0.86	0.15	0.81	0.17
IFO	OGUN	11674	11487	0.91	0.09	0.85	0.12	0.78	0.13
IGABI	KADUNA	24999	21847	0.77	0.20	0.62	0.23	0.58	0.22
IGALAMELA-ODOLU	KOGI	4225	5179	0.88	0.14	0.77	0.21	0.69	0.22
IGBO-ETITI	ENUGU	2008	2884	0.94	0.09	0.87	0.15	0.82	0.15
IGBO-EZE-NORTH	ENUGU	4047	5483	0.90	0.13	0.81	0.21	0.76	0.22
IGBO-EZE-SOUTH	ENUGU	2400	3465	0.91	0.13	0.81	0.21	0.76	0.21
IGUEGBEN	EDO	803	1326	0.93	0.11	0.89	0.15	0.84	0.16
IHIALA	ANAMBRA	5613	5975	0.90	0.11	0.85	0.13	0.81	0.13
IHITTE/UBOMA	IMO	1417	1797	0.93	0.09	0.87	0.13	0.79	0.16
IJEBU EAST	OGUN	1927	2814	0.90	0.14	0.83	0.19	0.73	0.23
IJEBU NORTH	OGUN	3952	6015	0.92	0.12	0.85	0.18	0.73	0.22
IJEBU NORTH EAST	OGUN	712	1127	0.92	0.12	0.85	0.20	0.74	0.23
IJEBU ODE	OGUN	1798	2748	0.93	0.11	0.86	0.19	0.77	0.22
IJERO	EKITI	2350	3155	0.94	0.07	0.92	0.09	0.88	0.11
IJUMU	KOGI	2115	3348	0.94	0.10	0.87	0.16	0.80	0.18
IKA	AKWA IBOM	674	952	0.94	0.09	0.88	0.14	0.80	0.16
IKA NORTH EAST	DELTA	2212	3996	0.92	0.14	0.89	0.16	0.84	0.17

IKA SOUTH	DELTA	1760	2965	0.93	0.12	0.89	0.16	0.84	0.17
IKARA	KADUNA	25081	13334	0.45	0.29	0.33	0.24	0.29	0.21
IKEDURU	IMO	2363	2768	0.91	0.10	0.86	0.13	0.79	0.14
IKEJA	LAGOS	5923	7505	0.93	0.09	0.89	0.11	0.84	0.13
IKENNE	OGUN	1562	2244	0.93	0.11	0.86	0.17	0.76	0.20
IKERE	EKITI	1343	1875	0.95	0.07	0.93	0.09	0.89	0.10
IKOLE	EKITI	1640	2281	0.95	0.07	0.92	0.11	0.87	0.13
IKOM	CROSS RIVER	1943	3375	0.94	0.11	0.88	0.17	0.69	0.26
IKONO	AKWA IBOM	1819	2247	0.92	0.10	0.83	0.16	0.73	0.18
IKORODU	LAGOS	5824	8329	0.94	0.09	0.88	0.14	0.82	0.15
IKOT ABASI	AKWA IBOM	2363	3123	0.91	0.12	0.82	0.17	0.74	0.19
IKOT EKPENE	AKWA IBOM	1427	1929	0.93	0.09	0.86	0.15	0.77	0.18
IKPOBA-OKHA	EDO	3353	5164	0.93	0.11	0.87	0.14	0.83	0.15
IKWERRE	RIVERS	3870	4238	0.87	0.14	0.83	0.15	0.76	0.17
IKWO	EBONYI	2073	3111	0.96	0.06	0.93	0.09	0.80	0.16
IKWUANO	ABIA	1390	1884	0.94	0.08	0.88	0.12	0.78	0.16
ILA	OSUN	895	1146	0.92	0.10	0.89	0.12	0.84	0.13
ILAJE	ONDO	9175	13258	0.80	0.29	0.75	0.29	0.69	0.30
ILE OLUJI/OKEIGBO	ONDO	2839	4112	0.93	0.09	0.91	0.11	0.85	0.13
ILEJEMEJE	EKITI	368	513	0.94	0.08	0.92	0.10	0.88	0.12
ILESHA EAST	OSUN	1221	1911	0.94	0.09	0.92	0.10	0.86	0.13
ILESHA WEST	OSUN	937	1504	0.94	0.09	0.92	0.11	0.85	0.15
ILLELA	SOKOTO	39877	15782	0.25	0.30	0.17	0.23	0.13	0.18
ILORIN EAST	KWARA	8957	11216	0.89	0.13	0.84	0.16	0.80	0.17
ILORIN SOUTH	KWARA	3692	4816	0.90	0.14	0.84	0.16	0.81	0.16
ILORIN WEST	KWARA	2717	3425	0.89	0.14	0.83	0.17	0.79	0.19

IMEKO AFON	OGUN	4129	6901	0.79	0.34	0.70	0.37	0.56	0.38
INGAWA	KATSINA	29230	11181	0.37	0.24	0.31	0.22	0.26	0.20
INI	AKWA IBOM	1021	1379	0.93	0.09	0.86	0.15	0.75	0.18
IPOKIA	OGUN	5528	7044	0.80	0.25	0.72	0.29	0.62	0.29
IRELE	ONDO	4865	6950	0.83	0.24	0.79	0.25	0.74	0.26
IREPO	OYO	19400	8207	0.23	0.33	0.18	0.27	0.15	0.23
IREPODUN	KWARA	2676	3032	0.91	0.10	0.87	0.13	0.83	0.14
IREPODUN	OSUN	1782	2453	0.90	0.13	0.87	0.16	0.80	0.19
IREPODUN/IFELODUN	EKITI	1036	1334	0.95	0.06	0.93	0.08	0.88	0.10
IREWOLE	OSUN	1520	2313	0.92	0.13	0.84	0.17	0.72	0.22
ISA	SOKOTO	31830	8829	0.18	0.23	0.14	0.20	0.11	0.16
ISE/ORUN	EKITI	1084	1569	0.95	0.07	0.93	0.08	0.88	0.11
ISEYIN	OYO	8483	11010	0.82	0.23	0.77	0.25	0.61	0.28
ISHIELU	EBONYI	1080	1576	0.96	0.06	0.91	0.11	0.85	0.13
ISI-UZO	ENUGU	1453	2219	0.94	0.08	0.89	0.14	0.82	0.17
ISIALA-NGWA NORTH	ABIA	1659	2013	0.93	0.08	0.89	0.11	0.79	0.13
ISIALA-NGWA SOUTH	ABIA	1667	2145	0.93	0.08	0.89	0.12	0.79	0.15
ISIALA MBANO	IMO	3883	4231	0.91	0.09	0.85	0.12	0.79	0.13
ISIN	KWARA	1044	1317	0.90	0.12	0.87	0.15	0.82	0.16
ISOKAN	OSUN	1905	2817	0.91	0.13	0.83	0.17	0.73	0.21
ISOKO NORTH	DELTA	2603	3322	0.90	0.13	0.86	0.14	0.79	0.16
ISOKO SOUTH	DELTA	5179	6169	0.88	0.14	0.84	0.15	0.77	0.17
ISU	IMO	2226	2643	0.90	0.11	0.84	0.14	0.80	0.15
ISUIKWUATO	ABIA	1176	1530	0.94	0.08	0.86	0.15	0.76	0.18
ITAS/GADAU	BAUCHI	30954	11368	0.36	0.24	0.26	0.20	0.21	0.16
ITESIWAJU	OYO	7207	8426	0.72	0.33	0.67	0.33	0.52	0.33
ITU	AKWA IBOM	2410	2761	0.87	0.15	0.76	0.21	0.64	0.24

IVO	EBONYI	786	1136	0.96	0.06	0.89	0.11	0.83	0.14
IWAJOWA	OYO	5497	7693	0.75	0.35	0.68	0.36	0.51	0.34
IWO	OSUN	2234	3252	0.92	0.12	0.86	0.16	0.73	0.23
IZZI	EBONYI	3031	3998	0.94	0.08	0.90	0.10	0.77	0.15
JABA	KADUNA	1773	3082	0.94	0.11	0.80	0.26	0.74	0.27
JADA	ADAMAWA	11446	13285	0.78	0.25	0.69	0.26	0.58	0.26
JAHUN	JIGAWA	27543	15002	0.51	0.27	0.37	0.23	0.30	0.20
JAKUSKO	YOBE	44073	15619	0.28	0.26	0.23	0.21	0.17	0.17
JALINGO	TARABA	2244	3210	0.86	0.20	0.79	0.24	0.66	0.27
JAMA'ARE	BAUCHI	15047	5526	0.32	0.25	0.24	0.21	0.20	0.19
JEGA	KEBBI	45466	10609	0.20	0.19	0.12	0.13	0.09	0.11
JEMA'A	KADUNA	3928	6627	0.94	0.10	0.81	0.21	0.74	0.23
JERE	BORNO	52108	34623	0.45	0.37	0.39	0.32	0.24	0.26
JIBIA	KATSINA	25888	11669	0.31	0.31	0.27	0.29	0.21	0.23
JOS EAST	PLATEAU	1085	1620	0.94	0.09	0.89	0.13	0.78	0.19
JOS NORTH	PLATEAU	2494	4389	0.96	0.07	0.92	0.11	0.84	0.16
JOS SOUTH	PLATEAU	2286	4512	0.97	0.06	0.92	0.12	0.85	0.16
KABBA/BUNU	KOGI	2513	3726	0.90	0.15	0.81	0.22	0.73	0.23
KABO	KANO	21308	11181	0.50	0.26	0.42	0.24	0.37	0.22
KACHIA	KADUNA	7470	8745	0.87	0.16	0.74	0.23	0.69	0.23
KADUNA NORTH	KADUNA	6783	10987	0.91	0.15	0.74	0.29	0.71	0.28
KADUNA SOUTH	KADUNA	3335	5323	0.91	0.14	0.73	0.31	0.70	0.31
KAFIN HAUSA	JIGAWA	34050	17606	0.46	0.28	0.32	0.25	0.25	0.21
KAFUR	KATSINA	27000	12928	0.50	0.24	0.44	0.24	0.39	0.21
KAGA	BORNO	16748	9273	0.31	0.38	0.26	0.34	0.18	0.25
KAGARKO	KADUNA	3541	4793	0.91	0.12	0.83	0.18	0.77	0.19
KAIAMA	KWARA	17739	7455	0.30	0.29	0.24	0.24	0.19	0.20
KAITA	KATSINA	30006	10440	0.25	0.26	0.21	0.23	0.17	0.19
KAJOLA	OYO	7605	10588	0.80	0.28	0.75	0.30	0.60	0.34

KAJURU	KADUNA	4509	6000	0.85	0.20	0.69	0.31	0.63	0.29
KALA/BALGE	BORNO	18962	16569	0.36	0.56	0.29	0.48	0.19	0.35
KALGO	KEBBI	27946	7229	0.23	0.20	0.13	0.14	0.10	0.11
KALTUNGO	GOMBE	8848	8158	0.75	0.23	0.67	0.25	0.58	0.24
KANAM	PLATEAU	10854	8967	0.68	0.27	0.61	0.26	0.53	0.24
KANKARA	KATSINA	38647	14696	0.32	0.26	0.29	0.23	0.25	0.22
KANKE	PLATEAU	2556	3252	0.86	0.17	0.81	0.19	0.70	0.22
KANKIA	KATSINA	21557	9256	0.36	0.28	0.31	0.24	0.27	0.22
KANO MUNICIPAL	KANO	12152	11812	0.78	0.21	0.72	0.23	0.67	0.24
KARASUWA	YOBE	14777	5928	0.31	0.28	0.26	0.25	0.18	0.20
KARAYE	KANO	20004	11320	0.50	0.28	0.41	0.26	0.35	0.23
KARIM LAMIDO	TARABA	7984	6873	0.76	0.20	0.69	0.22	0.56	0.22
KARU	NASARAWA	3284	3845	0.92	0.10	0.81	0.17	0.75	0.18
KATAGUM	BAUCHI	37460	12387	0.27	0.24	0.20	0.20	0.17	0.17
KATCHA	NIGER	14069	9378	0.53	0.31	0.46	0.30	0.39	0.27
KATSINA	KATSINA	43313	22245	0.36	0.33	0.31	0.30	0.27	0.26
KATSINA-ALA	BENUE	6668	7409	0.84	0.18	0.78	0.19	0.61	0.24
KAUGAMA	JIGAWA	11947	7850	0.56	0.29	0.40	0.27	0.30	0.22
KAURA	KADUNA	1920	3681	0.94	0.11	0.85	0.20	0.79	0.22
KAURA NAMODA	ZAMFARA	63363	10589	0.11	0.15	0.10	0.13	0.08	0.11
KAURU	KADUNA	12781	12032	0.75	0.23	0.63	0.23	0.56	0.22
KAZAURE	JIGAWA	15191	9021	0.52	0.28	0.42	0.27	0.36	0.24
KEANA	NASARAWA	2566	3205	0.86	0.18	0.74	0.25	0.63	0.25
KEBBE	SOKOTO	34223	7872	0.18	0.19	0.12	0.14	0.08	0.11
KEFFI	NASARAWA	2382	3390	0.87	0.19	0.69	0.32	0.64	0.30
KHANA	RIVERS	4574	6067	0.93	0.09	0.88	0.13	0.82	0.15
KIBIYA	KANO	20014	10914	0.45	0.30	0.35	0.27	0.30	0.23
KIRFI	BAUCHI	22915	9856	0.32	0.29	0.27	0.24	0.22	0.21
KIRI KASAMA	JIGAWA	21067	12412	0.47	0.31	0.35	0.29	0.26	0.23

KIRU	KANO	37780	19483	0.46	0.28	0.35	0.23	0.31	0.21
KIYAWA	JIGAWA	21573	8928	0.37	0.26	0.28	0.21	0.23	0.18
KOGI	KOGI	2992	4655	0.88	0.19	0.77	0.27	0.69	0.28
KOKO/BESSE	KEBBI	26415	8849	0.24	0.26	0.15	0.19	0.10	0.14
KOKONA	NASARAWA	2647	3230	0.89	0.13	0.74	0.22	0.66	0.21
KOLOKUMA/OPOKUMA	BAYELSA	2524	2264	0.80	0.18	0.74	0.18	0.65	0.19
KONDUGA	BORNO	18364	10730	0.44	0.33	0.37	0.28	0.23	0.21
KONSHISHA	BENUE	6652	7904	0.88	0.15	0.83	0.17	0.70	0.20
KONTAGORA	NIGER	25403	9484	0.25	0.28	0.18	0.23	0.14	0.18
KOSOFE	LAGOS	5436	7247	0.94	0.08	0.90	0.10	0.84	0.13
KUBAN	KADUNA	30908	16965	0.53	0.26	0.37	0.24	0.32	0.21
KUDAN	KADUNA	15603	8729	0.56	0.25	0.46	0.23	0.42	0.21
KUJE	FCT	3012	3566	0.93	0.09	0.85	0.15	0.76	0.17
KUKAWA	BORNO	34349	20486	0.29	0.42	0.23	0.34	0.14	0.24
KUMBOTSO	KANO	29890	23833	0.75	0.20	0.68	0.21	0.63	0.21
KUNCHI	KANO	16649	7456	0.42	0.26	0.35	0.23	0.29	0.20
KURA	KANO	13030	8604	0.60	0.26	0.51	0.25	0.46	0.23
KURFI	KATSINA	19872	7991	0.31	0.28	0.27	0.26	0.22	0.22
KURMI	TARABA	5525	6517	0.68	0.37	0.60	0.37	0.45	0.32
KUSADA	KATSINA	13823	6040	0.38	0.27	0.32	0.25	0.28	0.23
KWALI	FCT	4785	5377	0.88	0.14	0.81	0.16	0.71	0.18
KWAMI	GOMBE	41040	11990	0.28	0.21	0.20	0.17	0.17	0.14
KWANDE	BENUE	8134	10875	0.87	0.17	0.81	0.19	0.65	0.22
KWARE	SOKOTO	35411	7520	0.18	0.17	0.11	0.12	0.09	0.11
KWAYA KUSAR	BORNO	10896	5950	0.35	0.35	0.28	0.31	0.23	0.25
LAFIA	NASARAWA	7989	8933	0.89	0.13	0.76	0.19	0.67	0.19
LAGELU	OYO	2466	3415	0.93	0.10	0.83	0.15	0.69	0.20
LAGOS ISLAND	LAGOS	378	666	0.94	0.11	0.90	0.14	0.85	0.18
LAGOS MAINLAND	LAGOS	976	1679	0.94	0.11	0.90	0.13	0.85	0.17

LANGTANG NORTH	PLATEAU	4704	6047	0.82	0.23	0.76	0.23	0.65	0.24
LANGTANG SOUTH	PLATEAU	4447	6037	0.78	0.30	0.70	0.32	0.56	0.31
LAPAI	NIGER	7177	6830	0.76	0.23	0.67	0.25	0.58	0.25
LARMURDE	ADAMAWA	3689	4027	0.79	0.22	0.72	0.24	0.62	0.24
LAU	TARABA	3219	3891	0.84	0.20	0.78	0.21	0.62	0.25
LAVUN	NIGER	11022	6190	0.46	0.30	0.38	0.27	0.32	0.24
LERE	KADUNA	22614	21144	0.71	0.27	0.62	0.27	0.53	0.25
LOGO	BENUE	8334	8385	0.77	0.23	0.70	0.24	0.57	0.26
LOKOJA	KOGI	5212	6298	0.85	0.19	0.75	0.27	0.67	0.26
MACHINA	YOBE	7195	3541	0.45	0.27	0.36	0.24	0.24	0.19
MADAGALI	ADAMAWA	11757	15925	0.66	0.45	0.60	0.44	0.48	0.41
MADOBI	KANO	15755	9478	0.62	0.23	0.53	0.22	0.48	0.21
MAFA	BORNO	20912	12779	0.37	0.38	0.31	0.33	0.18	0.25
MAGAMA	NIGER	35290	14000	0.30	0.28	0.21	0.22	0.15	0.16
MAGUMERI	BORNO	21728	9560	0.26	0.33	0.21	0.29	0.13	0.20
MAI'ADUA	KATSINA	23946	12079	0.40	0.30	0.35	0.27	0.28	0.23
MAIDUGURI	BORNO	37700	27737	0.48	0.38	0.41	0.34	0.27	0.27
MAIGATARI	JIGAWA	12916	10352	0.55	0.36	0.44	0.32	0.33	0.28
MAIHA	ADAMAWA	10272	9862	0.60	0.38	0.52	0.36	0.42	0.32
MAIYAMA	KEBBI	19594	4650	0.20	0.19	0.11	0.15	0.08	0.11
MAKARFI	KADUNA	20401	12116	0.48	0.31	0.38	0.27	0.34	0.25
MAKODA	KANO	25915	14148	0.50	0.27	0.42	0.24	0.36	0.22
MALAM MADURI	JIGAWA	13586	8649	0.52	0.31	0.38	0.31	0.29	0.24
MALUMFASHI	KATSINA	21935	11755	0.48	0.28	0.43	0.27	0.38	0.25
MANGU	PLATEAU	3189	5159	0.95	0.09	0.89	0.13	0.78	0.18
MANI	KATSINA	29824	9746	0.30	0.23	0.25	0.20	0.21	0.17
MARADUN	ZAMFARA	55232	8237	0.11	0.13	0.09	0.11	0.07	0.09
MARIGA	NIGER	37417	11200	0.20	0.24	0.14	0.17	0.11	0.14
MAKURDI	BENUE	6216	7974	0.89	0.14	0.81	0.20	0.73	0.22

MARTE	BORNO	21938	15164	0.33	0.46	0.26	0.39	0.16	0.28
MARU	ZAMFARA	54305	10795	0.14	0.17	0.10	0.14	0.08	0.11
MASHEGU	NIGER	35841	15071	0.32	0.29	0.24	0.23	0.20	0.20
MASHI	KATSINA	28823	9956	0.27	0.25	0.23	0.22	0.18	0.18
MATAZU	KATSINA	14388	7073	0.39	0.30	0.35	0.27	0.30	0.25
MAYO-BELWA	ADAMAWA	3711	4713	0.83	0.21	0.75	0.24	0.62	0.24
MBATOLI	IMO	3974	4500	0.90	0.11	0.85	0.13	0.79	0.14
MBO	AKWA IBOM	2877	4046	0.86	0.20	0.72	0.28	0.60	0.28
MICHIKA	ADAMAWA	9515	13272	0.76	0.33	0.71	0.33	0.60	0.32
MIGA	JIGAWA	11943	7491	0.53	0.29	0.37	0.27	0.29	0.22
MIKANG	PLATEAU	2573	3437	0.88	0.16	0.82	0.19	0.68	0.23
MINJIBIR	KANO	20980	11753	0.58	0.24	0.50	0.23	0.44	0.20
MISAU	BAUCHI	44808	14766	0.26	0.24	0.20	0.21	0.17	0.17
MKPAT ENIN	AKWA IBOM	3215	3988	0.90	0.13	0.78	0.21	0.70	0.21
MOBA	EKITI	1632	2228	0.94	0.08	0.92	0.10	0.88	0.11
MOBBAR	BORNO	20360	8221	0.21	0.32	0.17	0.28	0.11	0.19
MOKWA	NIGER	27356	16627	0.56	0.27	0.45	0.25	0.38	0.22
MONGUNO	BORNO	18836	11042	0.30	0.41	0.24	0.35	0.15	0.25
MOPA-MURO	KOGI	750	1364	0.91	0.16	0.84	0.23	0.77	0.27
MORO	KWARA	4979	5265	0.79	0.22	0.71	0.23	0.65	0.23
MUBI NORTH	ADAMAWA	7709	8458	0.76	0.26	0.70	0.26	0.60	0.26
MUBI SOUTH	ADAMAWA	10306	10314	0.72	0.28	0.65	0.28	0.55	0.27
MUNICIPAL AREA COUNCIL	FCT	13986	17687	0.95	0.06	0.90	0.10	0.85	0.11
MUSAWA	KATSINA	22386	11913	0.43	0.30	0.38	0.28	0.33	0.25
MUSHIN	LAGOS	5955	8134	0.94	0.09	0.90	0.11	0.84	0.14
MUYA	NIGER	5628	5901	0.74	0.27	0.63	0.30	0.57	0.27

NAFADA	GOMBE	19889	5765	0.24	0.22	0.20	0.19	0.16	0.16
NANGERE	YOBE	9817	3180	0.31	0.22	0.26	0.21	0.21	0.18
NASARAWA	NASARAWA	5508	6620	0.86	0.17	0.73	0.24	0.66	0.23
NASARAWA EGON	NASARAWA	2239	2883	0.93	0.09	0.81	0.19	0.72	0.21
NASSARAWA	KANO	8129	7370	0.76	0.21	0.69	0.22	0.64	0.22
NDOKWA EAST	DELTA	2014	2224	0.90	0.11	0.85	0.13	0.79	0.15
NDOKWA WEST	DELTA	2221	2963	0.91	0.12	0.87	0.14	0.81	0.16
NEMBE	BAYELSA	5472	5086	0.71	0.27	0.66	0.28	0.59	0.26
NGALA	BORNO	30747	25938	0.35	0.55	0.29	0.47	0.19	0.34
NGANZAI	BORNO	18369	8636	0.26	0.35	0.21	0.30	0.13	0.20
NGASKI	KEBBI	23028	10005	0.33	0.29	0.23	0.23	0.15	0.16
NGOR-OKPALA	IMO	2359	2754	0.91	0.10	0.87	0.13	0.78	0.16
NGURU	YOBE	18726	10867	0.41	0.34	0.32	0.30	0.23	0.23
NINGI	BAUCHI	50010	20038	0.38	0.25	0.31	0.21	0.23	0.17
NJABA	IMO	2126	2330	0.90	0.11	0.84	0.13	0.79	0.14
NJIKOKA	ANAMBRA	1522	1607	0.93	0.07	0.90	0.09	0.87	0.11
NKANU EAST	ENUGU	916	1378	0.96	0.06	0.90	0.11	0.84	0.14
NKANU WEST	ENUGU	796	1198	0.96	0.06	0.90	0.12	0.85	0.13
NKWERRE	IMO	1096	1330	0.91	0.11	0.84	0.15	0.80	0.16
NNEWI NORTH	ANAMBRA	2079	2157	0.92	0.08	0.88	0.11	0.85	0.12
NNEWI SOUTH	ANAMBRA	4002	4015	0.92	0.08	0.87	0.10	0.84	0.11
NSIT ATAI	AKWA IBOM	1638	1960	0.88	0.14	0.75	0.21	0.64	0.22
NSIT IBOM	AKWA IBOM	2332	2689	0.90	0.12	0.77	0.18	0.68	0.21
NSIT UBIUM	AKWA IBOM	2527	3052	0.89	0.13	0.75	0.21	0.65	0.21
NSUKKA	ENUGU	4128	5617	0.92	0.11	0.83	0.18	0.78	0.18
NUMAN	ADAMAWA	2911	3436	0.86	0.17	0.79	0.20	0.69	0.22

NWANGELE	IMO	2218	2515	0.91	0.10	0.85	0.13	0.80	0.14
OBAFEMI OWODE	OGUN	4343	4494	0.90	0.10	0.82	0.15	0.72	0.17
OBANLIKU	CROSS RIVER	1899	3103	0.90	0.16	0.83	0.21	0.68	0.29
OBI	BENUE	2029	2992	0.90	0.14	0.86	0.17	0.75	0.21
OBI	NASARAWA	3940	5166	0.88	0.16	0.75	0.22	0.65	0.24
OBI NWGA	ABIA	1745	2095	0.94	0.07	0.89	0.11	0.81	0.13
OBIO/AKPOR	RIVERS	5650	6468	0.91	0.11	0.87	0.12	0.82	0.14
OBOKUN	OSUN	1923	2449	0.92	0.10	0.90	0.11	0.83	0.13
OBOT AKARA	AKWA IBOM	1627	2185	0.94	0.08	0.88	0.14	0.79	0.16
OBOWO	IMO	1748	2284	0.93	0.09	0.88	0.12	0.81	0.14
OBUBRA	CROSS RIVER	1449	2479	0.95	0.09	0.90	0.13	0.72	0.23
OBUDU	CROSS RIVER	2840	4618	0.91	0.14	0.84	0.21	0.69	0.28
ODEDA	OGUN	3222	3938	0.88	0.15	0.79	0.20	0.66	0.22
ODIGBO	ONDO	5986	8129	0.86	0.19	0.82	0.20	0.76	0.22
ODO OTIN	OSUN	2721	3361	0.89	0.13	0.85	0.15	0.80	0.16
ODOGBOLU	OGUN	1731	2488	0.93	0.10	0.85	0.18	0.75	0.23
ODUKPANI	CROSS RIVER	6864	7343	0.87	0.14	0.78	0.17	0.63	0.22
OFFA	KWARA	1553	1906	0.89	0.14	0.84	0.16	0.80	0.17
OFU	KOGI	6101	7290	0.85	0.18	0.75	0.22	0.67	0.24
OGBA/EGBEMA/NDONI	RIVERS	6827	7945	0.89	0.13	0.84	0.15	0.78	0.17
OGBADIBO	BENUE	2675	3762	0.91	0.13	0.85	0.18	0.78	0.19
OGBARU	ANAMBRA	5637	6390	0.91	0.10	0.88	0.11	0.84	0.12
OGBIA	BAYELSA	9586	7805	0.73	0.22	0.68	0.22	0.60	0.21
OGBOMOSHO NORTH	OYO	3752	5605	0.89	0.16	0.84	0.19	0.77	0.21
OGBOMOSHO SOUTH	OYO	1784	2767	0.89	0.17	0.85	0.20	0.76	0.24

OGO OLUWA	OYO	1020	1376	0.88	0.16	0.83	0.19	0.71	0.24
OGOJA	CROSS RIVER	1868	2837	0.94	0.09	0.88	0.16	0.71	0.23
OGORI/MAGONGO	KOGI	1147	1673	0.93	0.11	0.86	0.16	0.81	0.17
OGU BOLO	RIVERS	1268	1770	0.93	0.09	0.89	0.12	0.85	0.14
OGUN WATERSIDE	OGUN	2365	3696	0.86	0.22	0.80	0.25	0.72	0.27
OGUTA	IMO	2639	2986	0.89	0.13	0.83	0.15	0.77	0.16
OHAFIA	ABIA	1583	2148	0.95	0.06	0.89	0.11	0.77	0.17
OHAJI/EGBEMA	IMO	2941	3538	0.88	0.14	0.84	0.15	0.76	0.17
OHAOZARA	EBONYI	981	1301	0.96	0.05	0.92	0.09	0.84	0.12
OHAUKWU	EBONYI	1699	2483	0.95	0.07	0.91	0.11	0.84	0.14
OHIMINI	BENUE	1972	2824	0.88	0.17	0.84	0.19	0.76	0.20
OJI-RIVER	ENUGU	1168	1500	0.95	0.07	0.87	0.13	0.83	0.13
OJO	LAGOS	8793	9973	0.91	0.11	0.86	0.13	0.81	0.15
OJU	BENUE	3625	4601	0.92	0.11	0.87	0.13	0.74	0.20
OKE ERO	KWARA	760	1063	0.93	0.10	0.90	0.12	0.86	0.13
OKEHI	KOGI	2077	2936	0.91	0.13	0.81	0.19	0.74	0.20
OKENE	KOGI	5148	6953	0.91	0.12	0.83	0.17	0.77	0.19
OKIGWE	IMO	2025	2422	0.93	0.09	0.84	0.15	0.77	0.17
OKITIPUPA	ONDO	7139	11300	0.84	0.26	0.79	0.27	0.73	0.28
OKOBO	AKWA IBOM	2683	3170	0.88	0.14	0.75	0.22	0.63	0.23
OKPE	DELTA	2371	3043	0.91	0.12	0.86	0.15	0.80	0.16
OKPOKWU	BENUE	3421	4700	0.91	0.13	0.86	0.16	0.79	0.19
OKRIKA	RIVERS	3457	4330	0.93	0.09	0.88	0.12	0.84	0.13
OLA OLUWA	OSUN	1340	1885	0.90	0.14	0.85	0.17	0.72	0.23
OLAMABORO	KOGI	3647	4751	0.88	0.15	0.81	0.19	0.74	0.21
OLORUNDA	OSUN	1554	2018	0.91	0.12	0.87	0.15	0.81	0.17
OLORUNSOGO	OYO	7409	6885	0.53	0.44	0.46	0.39	0.40	0.35

OLUYOLE	OYO	2425	3396	0.92	0.11	0.82	0.19	0.69	0.22
OMALA	KOGI	3578	5367	0.84	0.24	0.76	0.28	0.68	0.27
OMUMMA	RIVERS	707	862	0.92	0.10	0.88	0.12	0.80	0.15
ONA ARA	OYO	3398	4701	0.92	0.11	0.81	0.19	0.68	0.22
ONDO EAST	ONDO	1195	1927	0.92	0.12	0.89	0.14	0.83	0.16
ONDO WEST	ONDO	5003	7095	0.91	0.13	0.87	0.14	0.82	0.18
ONICHA	EBONYI	1719	2286	0.96	0.05	0.92	0.08	0.84	0.11
ONITSHA NORTH	ANAMBRA	1058	1338	0.92	0.10	0.89	0.12	0.87	0.13
ONITSHA SOUTH	ANAMBRA	352	627	0.92	0.14	0.89	0.17	0.87	0.19
ONNA	AKWA IBOM	2390	3311	0.90	0.14	0.75	0.25	0.67	0.25
OPOBO/NKORO	RIVERS	145	261	0.93	0.13	0.88	0.17	0.82	0.21
OREDO	EDO	4173	6821	0.93	0.11	0.87	0.15	0.84	0.16
ORELOPE	OYO	15202	7160	0.24	0.36	0.20	0.30	0.17	0.25
ORHIONMWON	EDO	2876	3865	0.92	0.10	0.88	0.13	0.82	0.14
ORI IRE	OYO	6272	7537	0.82	0.21	0.77	0.22	0.67	0.25
ORIADE	OSUN	1405	1920	0.94	0.08	0.92	0.09	0.85	0.12
ORLU	IMO	2469	2560	0.90	0.10	0.83	0.14	0.79	0.15
OROLU	OSUN	935	1284	0.90	0.14	0.86	0.16	0.79	0.19
ORON	AKWA IBOM	2047	2920	0.88	0.17	0.73	0.25	0.62	0.28
ORSU	IMO	976	1014	0.90	0.11	0.83	0.15	0.79	0.15
ORU EAST	IMO	2185	2176	0.89	0.11	0.83	0.14	0.77	0.15
ORU WEST	IMO	2234	2445	0.89	0.12	0.83	0.14	0.79	0.15
ORUK ANAM	AKWA IBOM	2859	3460	0.92	0.10	0.83	0.16	0.75	0.17
ORUMBA NORTH	ANAMBRA	1072	1181	0.93	0.07	0.87	0.12	0.83	0.13
ORUMBA SOUTH	ANAMBRA	1570	1754	0.93	0.08	0.86	0.13	0.81	0.14
OSE	ONDO	1492	2118	0.94	0.08	0.90	0.11	0.85	0.13

OSHIMILI NORTH	DELTA	1964	2311	0.91	0.11	0.87	0.13	0.83	0.14
OSHIMILI SOUTH	DELTA	1706	1948	0.92	0.09	0.89	0.11	0.85	0.12
OSHODI/ISOLO	LAGOS	6974	8395	0.93	0.08	0.90	0.10	0.84	0.13
OSISIOMA NGWA	ABIA	2455	3129	0.93	0.09	0.90	0.11	0.82	0.13
OSOGBO	OSUN	2582	3776	0.92	0.12	0.88	0.15	0.82	0.17
OTURKPO	BENUE	5641	7589	0.88	0.16	0.84	0.17	0.76	0.18
OVIA NORTH-EAST	EDO	2593	3734	0.92	0.11	0.87	0.13	0.82	0.15
OVIA SOUTH-WEST	EDO	2770	4025	0.89	0.17	0.84	0.18	0.79	0.20
OWAN EAST	EDO	1643	2361	0.94	0.08	0.89	0.12	0.84	0.14
OWAN WEST	EDO	987	1487	0.94	0.09	0.90	0.12	0.86	0.14
OWERRI MUNICIPAL	IMO	1566	2139	0.91	0.13	0.86	0.17	0.80	0.17
OWERRI NORTH	IMO	2624	3216	0.91	0.11	0.86	0.13	0.80	0.14
OWERRI WEST	IMO	1222	1558	0.89	0.14	0.84	0.16	0.77	0.17
OWO	ONDO	2216	3660	0.95	0.09	0.92	0.10	0.87	0.12
OYE	EKITI	1245	1731	0.95	0.07	0.92	0.10	0.88	0.11
OYI	ANAMBRA	1546	1745	0.93	0.08	0.89	0.10	0.87	0.11
OYIGBO	RIVERS	1144	1408	0.93	0.09	0.88	0.11	0.82	0.13
OYO EAST	OYO	2453	3633	0.89	0.16	0.84	0.19	0.70	0.24
OYO WEST	OYO	3183	4411	0.88	0.16	0.83	0.19	0.68	0.24
OYUN	KWARA	2174	2570	0.89	0.13	0.84	0.15	0.80	0.16
PAIKORO	NIGER	8347	7425	0.77	0.21	0.67	0.23	0.59	0.23
PANKSHIN	PLATEAU	3334	5005	0.92	0.12	0.86	0.16	0.74	0.20
PATANI	DELTA	2421	2555	0.81	0.20	0.76	0.20	0.66	0.21
PATEGI	KWARA	10859	8936	0.58	0.35	0.49	0.33	0.43	0.30
PORT-HARCOURT	RIVERS	4837	5966	0.92	0.10	0.88	0.13	0.84	0.14
POTISKUM	YOBE	34000	16512	0.38	0.30	0.33	0.29	0.28	0.25
QUA'AN PAN	PLATEAU	5007	6844	0.89	0.15	0.80	0.20	0.67	0.22
RABAH	SOKOTO	29639	5566	0.14	0.16	0.08	0.12	0.07	0.10
RAFI	NIGER	26190	15209	0.41	0.34	0.34	0.30	0.30	0.27

RANO	KANO	23021	11995	0.45	0.29	0.34	0.25	0.30	0.23
REMO NORTH	OGUN	923	1340	0.92	0.12	0.84	0.18	0.73	0.22
RIJAU	NIGER	31720	11767	0.27	0.27	0.16	0.21	0.11	0.16
RIMI	KATSINA	24895	8563	0.30	0.24	0.25	0.22	0.21	0.19
RIMIN GADO	KANO	12213	6713	0.54	0.25	0.46	0.24	0.41	0.23
RINGIM	JIGAWA	19446	12270	0.59	0.26	0.48	0.25	0.41	0.23
RIYOM	PLATEAU	1244	2357	0.96	0.08	0.89	0.17	0.80	0.22
ROGO	KANO	27047	14914	0.50	0.27	0.43	0.25	0.38	0.23
RONI	JIGAWA	13111	6588	0.46	0.27	0.37	0.24	0.31	0.22
SABON BIRNI	SOKOTO	40308	14509	0.21	0.29	0.16	0.24	0.12	0.19
SABON GARI	KADUNA	18225	13640	0.67	0.25	0.55	0.25	0.51	0.24
SABUWA	KATSINA	21439	9697	0.29	0.32	0.24	0.28	0.21	0.25
SAFANA	KATSINA	32064	13069	0.28	0.29	0.24	0.26	0.20	0.22
SAGBAMA	BAYELSA	7095	5588	0.80	0.16	0.74	0.16	0.65	0.17
SAKABA	KEBBI	16112	6117	0.22	0.30	0.12	0.19	0.09	0.15
SAKI EAST	OYO	13275	5805	0.26	0.32	0.22	0.29	0.17	0.23
SAKI WEST	OYO	30355	13708	0.27	0.33	0.23	0.29	0.18	0.24
SANDAMU	KATSINA	15288	8699	0.50	0.28	0.44	0.26	0.36	0.24
SANGA	KADUNA	1819	3081	0.95	0.09	0.83	0.23	0.74	0.25
SAPELE	DELTA	3265	5346	0.91	0.15	0.86	0.18	0.81	0.18
SARDAUNA	TARABA	12611	14407	0.74	0.30	0.65	0.30	0.48	0.29
SHAGAMU	OGUN	3508	4650	0.93	0.10	0.86	0.15	0.77	0.17
SHAGARI	SOKOTO	35220	6434	0.14	0.16	0.09	0.12	0.07	0.09
SHANGA	KEBBI	25576	10433	0.29	0.29	0.18	0.22	0.11	0.16
SHANI	BORNO	15884	10078	0.44	0.35	0.38	0.32	0.31	0.27
SHANONO	KANO	18881	10424	0.47	0.29	0.41	0.26	0.35	0.22
SHELLENG	ADAMAWA	15391	10797	0.57	0.30	0.51	0.28	0.41	0.25
SHENDAM	PLATEAU	6366	9000	0.86	0.19	0.79	0.20	0.65	0.24
SHINKAFI	ZAMFARA	22586	5628	0.17	0.21	0.14	0.19	0.11	0.15

SHIRA	BAUCHI	46676	14806	0.27	0.23	0.21	0.19	0.17	0.17
SHIRORO	NIGER	20838	15996	0.62	0.29	0.52	0.27	0.46	0.25
SHOMGOM	GOMBE	3833	4152	0.83	0.18	0.75	0.20	0.64	0.22
SHOMOLU	LAGOS	657	1302	0.94	0.13	0.90	0.16	0.84	0.23
SILAME	SOKOTO	23061	6055	0.19	0.21	0.13	0.17	0.10	0.13
SOBA	KADUNA	33990	17289	0.51	0.25	0.37	0.23	0.33	0.20
SOKOTO NORTH	SOKOTO	24810	7427	0.21	0.24	0.13	0.19	0.12	0.16
SOKOTO SOUTH	SOKOTO	32316	9828	0.21	0.24	0.13	0.19	0.11	0.16
SONG	ADAMAWA	15862	12552	0.63	0.29	0.56	0.28	0.45	0.25
SOUTHERN IJAW	BAYELSA	19609	15946	0.69	0.26	0.63	0.25	0.54	0.24
SULE TANKAKAR	JIGAWA	9708	7898	0.61	0.31	0.51	0.30	0.41	0.27
SULEJA	NIGER	3333	4177	0.93	0.09	0.88	0.13	0.84	0.14
SUMAILA	KANO	41221	20791	0.41	0.30	0.34	0.25	0.28	0.21
SURU	KEBBI	30334	8264	0.21	0.21	0.13	0.15	0.09	0.12
SURULERE	LAGOS	3873	5481	0.94	0.09	0.90	0.12	0.85	0.16
SURULERE	OYO	3187	4243	0.88	0.16	0.84	0.17	0.75	0.20
TAFA	NIGER	1263	1636	0.93	0.10	0.88	0.13	0.83	0.15
TAFAWA-BALEWA	BAUCHI	13884	11401	0.74	0.22	0.68	0.22	0.59	0.22
TAI	RIVERS	1870	2506	0.93	0.09	0.89	0.12	0.84	0.14
TAKAI	KANO	25605	10989	0.40	0.26	0.34	0.24	0.27	0.21
TAKUM	TARABA	5978	7507	0.82	0.22	0.78	0.22	0.62	0.24
TALATA MAFARA	ZAMFARA	45267	6541	0.10	0.13	0.08	0.11	0.06	0.09
TAMBUWAL	SOKOTO	37347	7466	0.16	0.17	0.11	0.12	0.08	0.10
TANGAZA	SOKOTO	21075	7032	0.22	0.26	0.14	0.19	0.11	0.16
TARAUNI	KANO	9255	8759	0.78	0.21	0.71	0.22	0.66	0.22
TARKA	BENUE	2971	3406	0.81	0.22	0.74	0.24	0.65	0.24
TARMUA	YOBE	14486	6677	0.28	0.33	0.24	0.30	0.17	0.23
TAURA	JIGAWA	11629	8339	0.60	0.29	0.47	0.27	0.39	0.23
TEUNGO	ADAMAWA	3590	4458	0.71	0.36	0.62	0.35	0.51	0.32

TOFA	KANO	13035	7597	0.61	0.23	0.54	0.23	0.48	0.21
TORO	BAUCHI	31231	18437	0.60	0.23	0.54	0.23	0.43	0.19
TOTO	NASARAWA	3160	4171	0.89	0.15	0.76	0.24	0.68	0.25
TSAFE	ZAMFARA	42471	10576	0.18	0.20	0.15	0.18	0.13	0.16
TSANYAWA	KANO	23604	11914	0.43	0.29	0.38	0.26	0.32	0.23
TUDUN WADA	KANO	33887	15555	0.43	0.26	0.31	0.21	0.27	0.19
TURETA	SOKOTO	24888	4036	0.12	0.14	0.07	0.10	0.06	0.08
UDENU	ENUGU	2059	2908	0.92	0.11	0.85	0.17	0.80	0.18
UDI	ENUGU	2107	2977	0.95	0.07	0.87	0.13	0.83	0.14
UDU	DELTA	2617	3299	0.89	0.13	0.84	0.15	0.80	0.16
UDUNG UKO	AKWA IBOM	1178	1641	0.87	0.18	0.72	0.27	0.60	0.28
UGHELLI NORTH	DELTA	6536	7503	0.89	0.13	0.84	0.14	0.78	0.16
UGHELLI SOUTH	DELTA	6241	6651	0.86	0.15	0.81	0.16	0.74	0.17
UGWUNAGBO	ABIA	1231	1650	0.93	0.09	0.89	0.11	0.82	0.15
UHUNMWONDE	EDO	1173	1837	0.94	0.10	0.88	0.14	0.83	0.16
UKANAFUN	AKWA IBOM	1444	1985	0.93	0.09	0.86	0.15	0.78	0.16
UKUM	BENUE	8286	9561	0.83	0.20	0.77	0.21	0.62	0.24
UKWA EAST	ABIA	920	1225	0.93	0.09	0.89	0.12	0.81	0.15
UKWA WEST	ABIA	1162	1376	0.92	0.09	0.89	0.11	0.81	0.14
UKWUANI	DELTA	1538	2295	0.91	0.13	0.87	0.15	0.82	0.16
UMU-NNEOCHI	ABIA	1488	1973	0.94	0.08	0.85	0.16	0.79	0.18
UMUAHIA NORTH	ABIA	1657	2286	0.94	0.09	0.87	0.13	0.77	0.16
UMUAHIA SOUTH	ABIA	1525	2071	0.94	0.09	0.88	0.13	0.78	0.15
UNGONGO	KANO	35625	25840	0.73	0.19	0.66	0.20	0.61	0.19
UNUIMO	IMO	1611	1862	0.92	0.10	0.84	0.13	0.78	0.14
URUAN	AKWA IBOM	2804	3235	0.87	0.15	0.76	0.21	0.62	0.22

URUE OFFONG/ORUKO	AKWA IBOM	1941	2597	0.87	0.17	0.72	0.25	0.61	0.26
USHONGO	BENUE	4816	5551	0.85	0.17	0.79	0.19	0.64	0.23
USSA	TARABA	2186	3125	0.81	0.27	0.76	0.26	0.59	0.28
UVWIE	DELTA	2558	3532	0.91	0.12	0.86	0.16	0.82	0.16
UYO	AKWA IBOM	5328	6026	0.89	0.13	0.77	0.21	0.67	0.22
UZO-UWANI	ENUGU	2101	2738	0.91	0.12	0.79	0.21	0.74	0.21
VANDEIKYA	BENUE	7161	9637	0.88	0.17	0.81	0.19	0.67	0.22
WAMAKO	SOKOTO	22092	4534	0.17	0.17	0.10	0.12	0.08	0.11
WAMBA	NASARAWA	960	1627	0.94	0.11	0.81	0.25	0.71	0.26
WARAWA	KANO	11143	6723	0.61	0.23	0.53	0.23	0.47	0.22
WARJI	BAUCHI	17651	8559	0.36	0.31	0.28	0.26	0.22	0.22
WARRI NORTH	DELTA	6509	8647	0.82	0.24	0.77	0.24	0.71	0.24
WARRI SOUTH	DELTA	6318	8102	0.90	0.13	0.85	0.17	0.80	0.17
WARRI SOUTH-WEST	DELTA	1696	2614	0.81	0.30	0.75	0.32	0.67	0.30
WASAGU/DANKO	KEBBI	56021	15764	0.18	0.23	0.11	0.17	0.08	0.13
WASE	PLATEAU	8884	7623	0.68	0.27	0.61	0.26	0.50	0.24
WUDIL	KANO	24552	11730	0.46	0.26	0.39	0.24	0.34	0.22
WUKARI	TARABA	12686	12517	0.78	0.22	0.72	0.22	0.58	0.24
WURNO	SOKOTO	37956	7154	0.16	0.16	0.09	0.12	0.08	0.10
WUSHISHI	NIGER	10090	7206	0.49	0.36	0.43	0.34	0.37	0.30
YABO	SOKOTO	18259	3766	0.16	0.17	0.11	0.13	0.08	0.11
YAGBA EAST	KOGI	2181	3757	0.91	0.15	0.85	0.19	0.79	0.22
YAGBA WEST	KOGI	3010	5254	0.89	0.20	0.83	0.24	0.77	0.25
YAKURR	CROSS RIVER	1753	2681	0.95	0.08	0.90	0.12	0.75	0.20
YALA	CROSS RIVER	2801	3630	0.93	0.09	0.88	0.12	0.72	0.19
YAMALTU/DEBA	GOMBE	63712	26525	0.41	0.25	0.31	0.22	0.27	0.21

YANKWASHI	JIGAWA	7667	4820	0.53	0.30	0.44	0.28	0.36	0.25
YAURI	KEBBI	12707	5922	0.33	0.31	0.23	0.25	0.14	0.18
YENEGOA	BAYELSA	15269	12599	0.78	0.18	0.73	0.18	0.66	0.18
YOLA NORTH	ADAMAWA	4649	6880	0.90	0.15	0.79	0.22	0.68	0.25
YOLA SOUTH	ADAMAWA	2502	3840	0.92	0.12	0.83	0.21	0.73	0.24
YORRO	TARABA	5223	6322	0.85	0.18	0.78	0.21	0.64	0.24
YUNUSARI	YOBE	21486	10078	0.27	0.34	0.23	0.30	0.15	0.23
YUSUFARI	YOBE	15807	7190	0.31	0.32	0.26	0.28	0.17	0.20
ZAKI	BAUCHI	24988	11437	0.38	0.28	0.29	0.25	0.23	0.20
ZANGO	KATSINA	15189	9454	0.51	0.30	0.45	0.29	0.36	0.25
ZANGON KATAF	KADUNA	4964	8018	0.92	0.12	0.81	0.22	0.74	0.22
ZARIA	KADUNA	32321	23838	0.64	0.26	0.52	0.26	0.48	0.24
ZING	TARABA	4353	5726	0.83	0.22	0.76	0.24	0.62	0.26
ZURMI	ZAMFARA	65612	13726	0.16	0.17	0.14	0.16	0.11	0.12
ZURU	KEBBI	30141	11475	0.22	0.30	0.14	0.22	0.10	0.18

*Estimates for this LGA are missing due to missing grid level values in one of the covariate rasters used for model-fitting.
